# Prenatal exposure to buprenorphine or methadone and adverse neurodevelopmental outcomes: population based cohort study

**DOI:** 10.1136/bmj-2025-087321

**Published:** 2026-04-15

**Authors:** Sabine Friedrich, Krista F Huybrechts, Loreen Straub, Sonia Hernandez-Diaz, Yanmin Zhu, Georg Hahn, Helen Mogun, Hendrée E Jones, Hilary S Connery, Jonathan M Davis, Kathryn J Gray, Barry Lester, Mishka Terplan, Brian T Bateman

**Affiliations:** 1 Division of Pharmacoepidemiology and Pharmacoeconomics, Department of Medicine, Brigham and Women’s Hospital and Harvard Medical School, Boston, MA 02120, USA; 2 Department of Epidemiology, Harvard T.H. Chan School of Public Health, Boston, MA 02115, USA; 3 UNC Horizons and Department of Obstetrics and Gynecology, University of North Carolina at Chapel Hill, Chapel Hill, NC, USA; 4 Division of Alcohol, Drugs, and Addiction, McLean Hospital and Department of Psychiatry, Harvard Medical School, Belmont, MA, USA; 5 Department of Pediatrics, Tufts Medical Center and the Tufts Clinical and Translational Science Institute, Boston, MA, USA; 6 Department of Obstetrics and Gynecology, University of Washington, Seattle, WA, USA; 7 Center for the Study of Children at Risk, Departments of Psychiatry and Pediatrics, Alpert Medical School of Brown University, and Women and Infants Hospital, Providence, RI, USA; 8 Friends Research Institute, Baltimore, MD, USA; 9 Department of Anesthesiology, Perioperative and Pain Medicine, Stanford University School of Medicine, Stanford, CA, USA

## Abstract

**Objective:**

To compare the incidence of neurodevelopmental disorders among children with prenatal exposure to buprenorphine versus methadone.

**Design:**

Population based cohort study.

**Setting:**

US nationwide Medicaid data on >2.5 million live births from 2000 to 2018.

**Participants:**

18 612 pregnancies exposed to buprenorphine or methadone, of which 587 were excluded from the analysis owing to exposure to the comparator drug.

**Main outcome measures:**

The primary outcome was a composite of neurodevelopmental disorders (autism spectrum disorder, attention deficit/hyperactivity disorder, developmental speech or language disorder, developmental coordination disorder, behavioural disorder, learning difficulty, or intellectual disability). Individual neurodevelopmental disorders were considered secondary outcomes. Cumulative incidences were obtained using Kaplan-Meier analyses, and hazard ratios using Cox proportional hazards regression. Propensity score overlap weighting was applied to adjust for confounding, including personal characteristics, maternal medical and mental health comorbidities, exposure to medications and other substances, proxies for severity of opioid use disorder, healthcare utilisation, and adequacy of prenatal care utilisation.

**Results:**

12 635 children were exposed to buprenorphine and 5390 to methadone prenatally. The crude cumulative incidence of any neurodevelopmental disorder at age 8 years among those exposed to buprenorphine was 34% (95% confidence interval (CI) 30% to 38%) and among those exposed to methadone was 33% (29% to 37%). Adjusted analyses suggested slightly lower hazards of any neurodevelopmental disorder associated with exposure to buprenorphine versus methadone (adjusted hazard ratio 0.81, 95% CI 0.70 to 0.94). Similar results were obtained for the individual neurodevelopmental disorders such as attention deficit/hyperactivity disorder (0.89, 0.65 to 1.21) and autism spectrum disorder (0.74, 0.46 to 1.21). With prevalent use, prenatal exposure to buprenorphine was associated with lower hazards of any neurodevelopmental disorder compared with prenatal exposure to methadone (adjusted hazard ratio 0.62, 0.51 to 0.76). This association was not observed with treatment initiation during pregnancy (adjusted hazard ratio 1.13, 0.90 to 1.42). Further sensitivity analyses indicated results consistent with no increased risk of neurodevelopmental disorders among pregnancies exposed to buprenorphine versus methadone.

**Conclusions:**

The findings of this study suggest no increased risk of long term adverse neurodevelopmental outcomes among children with prenatal exposure to buprenorphine versus methadone, further supporting buprenorphine as a safe treatment option for opioid use disorder during pregnancy.

## Introduction

Methadone and buprenorphine are recommended medications for the treatment of opioid use disorder (MOUD) during pregnancy.^1^ MOUD has been shown to improve maternal and fetal outcomes by promoting adherence to prenatal care and reducing the risk of maternal overdose and preterm birth.^2^

Methadone and buprenorphine differ in their pharmacology and they are administered differently in clinical practice. Methadone generally requires daily clinic visits, whereas buprenorphine can be dispensed as an outpatient prescription.^1^ Although methadone is a full mu receptor agonist, buprenorphine acts as a partial mu receptor agonist and a kappa receptor antagonist.^1^ Differences in treatment outcomes between these drugs have been observed in pregnancy that are potentially attributable to these factors. Although treatment retention may be lower for those treated with buprenorphine versus methadone,^3^ ^4^ ^5^ ^6^ recent evidence suggests more favourable neonatal outcomes with buprenorphine compared with methadone.^3^ ^7^ ^8^

A key consideration in selecting drugs for use in pregnancy is the impact on long term neurodevelopmental outcomes in the offspring. Currently, comparative information on the long term safety of methadone versus buprenorphine is limited. Preclinical data suggest that these drugs may have differential effects on the developing central nervous system, indicating potential pathways for poorer neurodevelopmental outcome with exposure to methadone versus buprenorphine.^9^ ^10^ ^11^ ^12^ Data in humans are mixed, with some studies suggesting better neurodevelopmental outcomes with exposure to buprenorphine^13^ ^14^ ^15^ ^16^ ^17^ and some suggesting comparable outcomes with both drugs.^16^ ^17^ ^18^ ^19^ Existing studies are, however, limited by short term follow-up (neonatal period, infancy, or early childhood) and small sample sizes.^13^ ^14^ ^15^ ^16^ ^17^ ^18^ ^19^

To address this important evidence gap, we utilised a large, US nationwide public health insurance database to compare prenatal exposure to buprenorphine with prenatal exposure to methadone with regards to the risk of neurodevelopmental disorders up to age 8 years, with robust control of confounders and various sensitivity analyses to examine potential sources of bias.

## Methods

### Data source and study population

The study cohort was nested in nationwide healthcare utilisation data for Medicaid insured pregnant individuals aged 12-55 years and their children in the US from 1 January 2000 to 31 December 2018. Derivation of the mother-child linked cohort is described in detail elsewhere.^20^ The data source included patient level information on personal characteristics; diagnoses and procedures recorded during emergency department, inpatient, and outpatient visits; and dispensing of prescription drugs to outpatients.

Pregnant individuals were required to have continuous Medicaid enrolment from 90 days before the day of their last menstrual period to 30 days after birth. Children were required to be enrolled in Medicaid from birth to at least three months after birth or until death if the child died within three months after birth. Follow-up of the child was from birth until diagnosis of the outcome, death, end of Medicaid coverage, or end of the study period (supplemental figure S1), whichever occurred first. We excluded pregnancies with a diagnosed chromosomal abnormality under the assumption that potential neurodevelopmental disorders in these children would unlikely be related to maternal use of MOUD.

### MOUD treatment

The exposure of interest was prenatal exposure to either buprenorphine or methadone between the last menstrual period and one day before birth. We excluded pregnancies with exposure to both drugs during pregnancy and pregnancies with exposure to the comparator drug during the three months before the start of pregnancy (supplemental figure S1).

Buprenorphine exposure included buprenorphine alone or in combination with naloxone, defined based on at least one drug dispensing or one qualifying Healthcare Common Procedure Coding System (HCPCS) code documented during the exposure window. Methadone exposure was defined based on at least one HCPCS code for administered methadone. We included only drug formulations and procedure codes indicated for opioid use disorder (supplemental table S1).

### Neurodevelopmental disorders

Outcomes of interest included autism spectrum disorder, attention deficit/hyperactivity disorder, developmental speech or language disorder, developmental coordination disorder, behavioural disorder, learning difficulty, and intellectual disability.^21^ ^22^ We used validated algorithms to assess diagnoses of neurodevelopmental disorders,^23^ with positive predictive values ranging from 82% to 98% (supplemental table S2). The minimum age from which the individual neurodevelopmental disorders were assessed differed based on diagnostic recommendations: any age for developmental coordination disorder, 1 year for autism spectrum disorder, 1.5 years for developmental speech or language disorder, and 2 years for all others.^23^ The primary outcome was the composite of any neurodevelopmental disorder, with time of event defined as timing of the first diagnosis of a neurodevelopmental disorder. The individual neurodevelopmental disorders were examined as secondary outcomes.

### Covariates

We considered a wide range of potential confounders and their proxies: personal characteristics; proxies for severity of opioid use disorder, including opioid related hospital admissions and emergency department visits; medical conditions associated with opioid use disorder (eg, sexually transmitted diseases, hepatitis B, hepatitis C, HIV, bacteraemia, sepsis, endocarditis); conditions describing non-opioid substance use or dependence; chronic pain disorders; mental health conditions; proxies for severity of mental health conditions; other comorbid conditions and obstetric comorbidity index^24^; concomitant drugs (including cumulative dose of prescription opioids, psychotropics); healthcare utilisation; and adequacy of prenatal care utilisation.^25^ Supplemental table S3 and supplemental figure S1 provide details of all covariates and their respective assessment windows.

### Main analyses

We used standardised differences to compare the balance of covariates between pregnancies exposed to buprenorphine and those exposed to methadone.^26^ Kaplan-Meier analyses were performed to obtain crude and adjusted cumulative incidences, with 95% confidence intervals (CIs) stratified by exposure status. As the number of children at risk with follow-up beyond age 8 years was sparse (supplemental figure S2) and most examined neurodevelopmental disorders are typically diagnosed before then, we applied administrative censoring after eight years of follow-up in all analyses.

Cox proportional hazards regression was used to estimate crude and adjusted hazard ratios, with buprenorphine defined as the exposed group and methadone as the reference group. To account for confounding, in adjusted analyses we applied propensity score overlap weighting accounting for all listed covariates.^27^ To achieve balance conditional on the time of treatment initiation, we estimated propensity score overlap weights within four subgroups according to the time of the first recorded exposure: within three months before the last menstrual period, during the first trimester, during the second trimester, and during the third trimester. To account for weighting, in all adjusted analyses we used robust variance estimation to obtain CIs.^28^ Analyses were performed in R, version 4.3.2, using the statistical packages “survival”, “survminer”, and “stats”.

### Sensitivity and subgroup analyses

To test the robustness of the findings and to inform interpretation of the results for the main analyses, we performed a range of sensitivity and subgroup analyses (supplemental table S4). Exposure during each trimester was analysed separately to examine whether findings differed based on timing of exposure. To minimise potential misclassification of exposure, we required more than one dispensing for buprenorphine in the assumption that if the drug was refilled, it was more likely to be taken as prescribed. To assess the possibility of residual confounding, we examined two negative control exposure windows: exposure within one year before but not during pregnancy, and initiation of exposure within nine months after delivery. We further performed additional adjustment for measures of socioeconomic status based on county level information (supplemental table S5). Owing to the potential for selection bias due to informative censoring (supplemental figure S2), we accounted for inverse probability of censoring weights^29^ in addition to propensity score overlap weights. To assess the possibility of differential surveillance by treatment, we evaluated gastroenteritis as a negative control outcome. Although gastroenteritis should not be causally related to exposure status, a difference in risk may reflect differences in healthcare seeking behaviour between individuals treated with buprenorphine or with methadone.

Subgroup analyses were performed in individuals with adequate or higher levels of prenatal care^25^ to minimise the risk of surveillance bias based on lack of access to healthcare, and in individuals with a recorded diagnosis of opioid use disorder to avoid misclassification of treatment indication. To further enhance comparability of the groups, we performed subgroup analysis including only pregnancies that occurred when both treatments were covered by Medicaid in the respective state.^8^

A history of treatment with MOUD before pregnancy (prevalent use) indicated established treatment, whereas treatment initiation during pregnancy (new use) might confer a higher risk of return to illicit opioid use or other substance use disorder. Moreover, awareness of pregnancy (which is more likely with treatment initiation during pregnancy) may affect treatment allocation towards buprenorphine or methadone. We therefore performed a sensitivity analysis stratified by prevalent treatment versus newly initiated treatment.

Finally, we explored potential differences in risk of neurodevelopmental disorders between those exposed to buprenorphine alone or in combination with naloxone compared with those exposed to methadone, excluding pregnancies with exposure to both buprenorphine formulations.

### Patient and public involvement

No patients or members of the public were directly involved in the conduct of this cohort study. However, interactions with affected populations in clinical care informed the study objectives.

## Results

**Fig 1:**
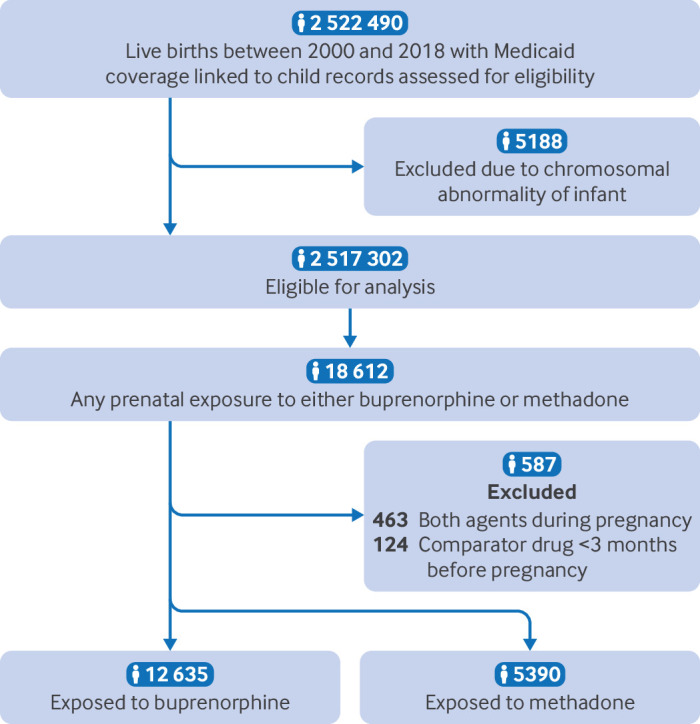
Study flowchart

A total of 12 635 pregnancies were exposed to buprenorphine and 5390 to methadone (fig 1). Mean maternal age was 28.3 years (standard deviation 4.6 years) for buprenorphine exposed pregnancies and 28.5 (4.9) years for methadone exposed pregnancies. A larger proportion of pregnant individuals in the buprenorphine group identified as white (87.8%) compared with those in the methadone group (75.2%). While tobacco and alcohol dependence and certain mental health conditions (eg, anxiety, depression) were more common among pregnant individuals in the buprenorphine group, most other characteristics were relatively well balanced between the two study groups before propensity score weighting (table 1). For adjusted analyses, propensity score overlap weighting resulted in a population that was balanced for all measured covariates by design (supplemental table S5).

**Table 1:** Maternal characteristics of pregnancies exposed to buprenorphine or methadone. Values are number (percentage) unless stated otherwise

Characteristics	Buprenorphine group (n=12 635)	Methadone group (n=5390)	Standardised difference*
Mean (SD) age (years)	28.26 (4.64)	28.47 (4.90)	−0.04
Race or ethnicity:			
White	11 092 (87.8)	4055 (75.2)	0.33
Black or African American	352 (2.8)	493 (9.1)	-0.27
Hispanic or Latino	438 (3.5)	406 (7.5)	-0.18
Asian or Pacific Islander	24 (0.2)	36 (0.7)	−0.07
Unknown or other	729 (5.8)	400 (7.4)	−0.07
Substance use or misuse:			
Alcohol misuse	959 (7.6)	249 (4.6)	0.12
Tobacco use	6156 (48.7)	2160 (40.1)	0.18
Comorbid other (non-opioid) substance use disorder	2510 (19.9)	860 (16.0)	0.10
Residential treatment for substance use disorder†	190 (1.5)	38 (0.7)	0.08
Medication assisted opioid use disorder treatment before pregnancy	7546 (59.7)	3094 (57.4)	0.05
Comorbidities:			
Pregestational hypertension	1008 (8.0)	466 (8.6)	−0.02
Pregestational diabetes	308 (2.4)	125 (2.3)	0.01
Chronic pain	1986 (15.7)	751 (13.9)	0.05
Back and neck pain	3015 (23.9)	1103 (20.5)	0.08
Obstetric comorbidity index‡:			
0-1	703 (5.6)	222 (4.1)	0.07
2	5152 (40.8)	2161 (40.1)	0.01
≥3	6780 (53.7)	3007 (55.8)	−0.04
Attention deficit/hyperactivity disorder	752 (6.0)	247 (4.6)	0.06
Anxiety disorder	3928 (31.1)	1259 (23.4)	0.17
Bipolar disorder	1486 (11.8)	557 (10.3)	0.05
Depression	4015 (31.8)	1281 (23.8)	0.18
Psychotic disorder	151 (1.2)	69 (1.3)	−0.01
Sleep disorder	617 (4.9)	188 (3.5)	0.07
Post-traumatic stress disorder	862 (6.8)	388 (7.2)	−0.02
Concomitant drugs:			
Oral antidiabetics or insulin	121 (1.0)	55 (1.0)	0.01
Antihypertensives	1458 (11.5)	414 (7.7)	0.13
Antidepressants	4986 (39.5)	1486 (27.6)	0.25
Antipsychotics	1371 (10.9)	465 (8.6)	0.08
Benzodiazepines	2417 (19.1)	936 (17.4)	0.05
Other anxiolytics or hypnotics	2469 (19.5)	742 (13.8)	0.16
Prescription opioids	3518 (27.8)	1778 (33.0)	-0.11
Healthcare utilisation:			
Opioid related emergency department visits	742 (5.9)	408 (7.6)	−0.07
Opioid related hospital admissions	889 (7.0)	332 (6.2)	0.04
Any hospital admission	1327 (10.5)	527 (9.8)	0.02
Median (IQR) No of emergency department visits	1 (0-2)	1 (0-3)	−0.06
Median (IQR) No of outpatient visits	6 (2-11)	3 (0-8)	0.15
Category of adequacy of prenatal care utilisation index§:			
Inadequate	5614 (44.4)	2504 (46.5)	−0.04
Intermediate	2087 (16.5)	910 (16.9)	−0.01
Adequate	1967 (15.6)	741 (13.7)	0.05
Adequate plus	2967 (23.5)	1235 (22.9)	0.01

See supplemental figure S1 and supplemental table S3 for assessment windows of different covariates.

IQR=interquartile range; SD=standard deviation.

* Measure of covariate balance between pregnancies exposed to buprenorphine versus methadone. In adjusted analyses, propensity score overlap weighting created a population that was balanced for all measured covariates by design (supplemental table S5).

† Includes inpatient and intensive outpatient treatment, including counselling and pharmacologic/detoxification, for alcohol or other substance dependence.

‡ See Bateman et al 2013.^24^

§ See Kotelchuck 1994.^25^

**Fig 2:**
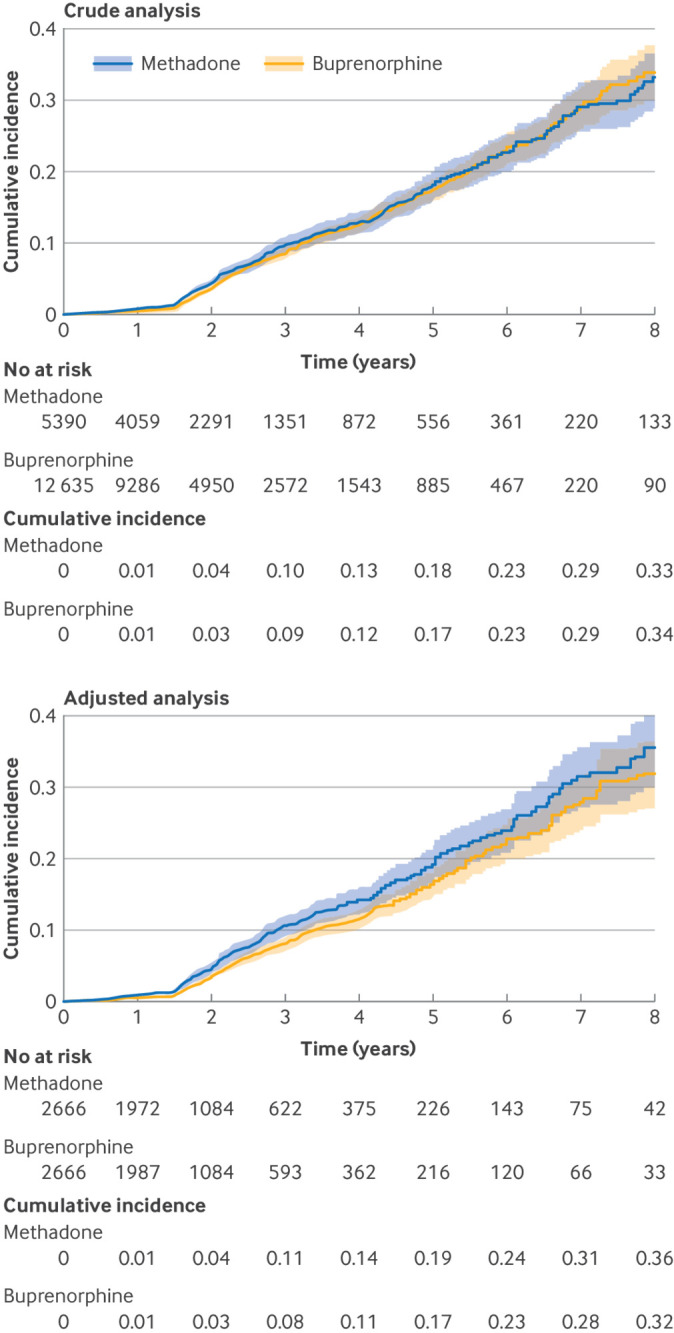
Crude and adjusted cumulative incidences of any neurodevelopmental disorders among children with prenatal exposure to methadone or buprenorphine as estimated using the Kaplan-Meier estimator across eight years of follow-up. Time of origin is delivery. Administrative censoring was applied after eight years. Number at risk represent children under observation and event-free at the respective follow-up time. For the adjusted analysis, the number at risk represents the sum of propensity score overlap weights in the respective exposure group

**Fig 3:**
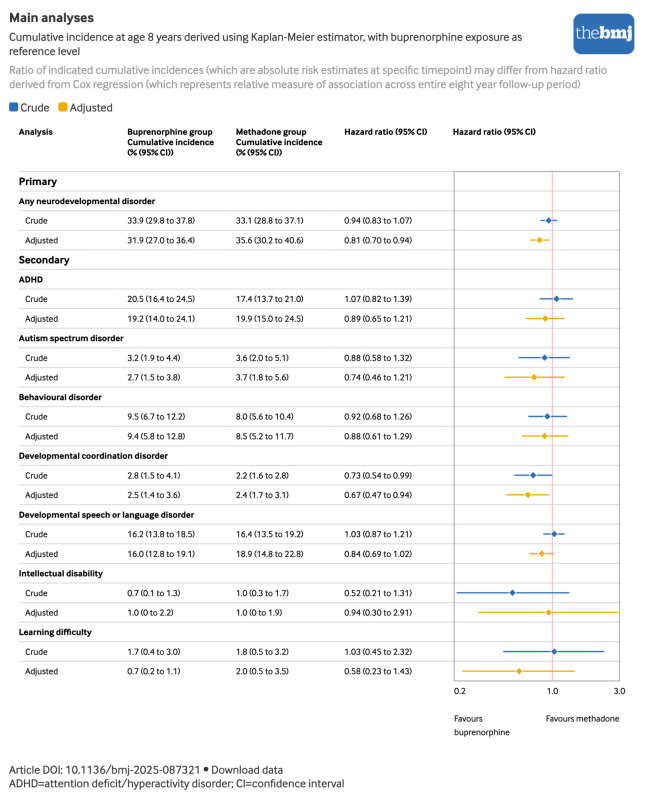
Main analyses. Cumulative incidences and hazard ratios of neurodevelopmental disorders among children with prenatal exposure to buprenorphine or methadone. An interactive version of this graphic is available at https://public.flourish.studio/visualisation/28043962/

The crude cumulative incidence of any neurodevelopmental disorder at age 8 years was 33.9% (95% CI 29.8% to 37.8%; 696 events) among children with prenatal exposure to buprenorphine and 33.1% (28.8% to 37.1%; 385 events) among children with prenatal exposure to methadone (fig 2). Attention deficit/hyperactivity disorder and developmental speech or language disorder were the most common neurodevelopmental disorders, with crude cumulative incidences at age 8 years of 20.5% (16.4% to 24.5%) and 16.2% (13.8% to 18.5%) in the buprenorphine group and 17.4% (13.7% to 21%) and 16.4% (13.5% to 19.2%) in the methadone group, respectively. Learning difficulties (<2%) and intellectual disabilities (<1%) were rare (fig 3). After adjustment, the cumulative incidence decreased slightly among children prenatally exposed to buprenorphine and increased slightly among children prenatally exposed to methadone (fig 2; supplemental figure S3).

Cox proportional hazards regression indicated a crude hazard ratio of 0.94 (95% CI 0.83 to 1.07) and an adjusted hazard ratio of 0.81 (95% CI 0.70 to 0.94) for the composite outcome of neurodevelopmental disorders overall. The adjusted hazard ratios for the individual neurodevelopmental disorders also indicated slightly lower hazards among children prenatally exposed to buprenorphine compared with those prenatally exposed to methadone, but the associations were imprecisely estimated owing to the small number of events. For example, the adjusted hazard ratio was 0.89 (0.65 to 1.21) for attention deficit/hyperactivity disorder, 0.84 (0.69 to 1.02) for developmental speech or language disorder, and 0.74 (0.46 to 1.21) for autism spectrum disorder (fig 3).

### Sensitivity and subgroup analyses

**Fig 4:**
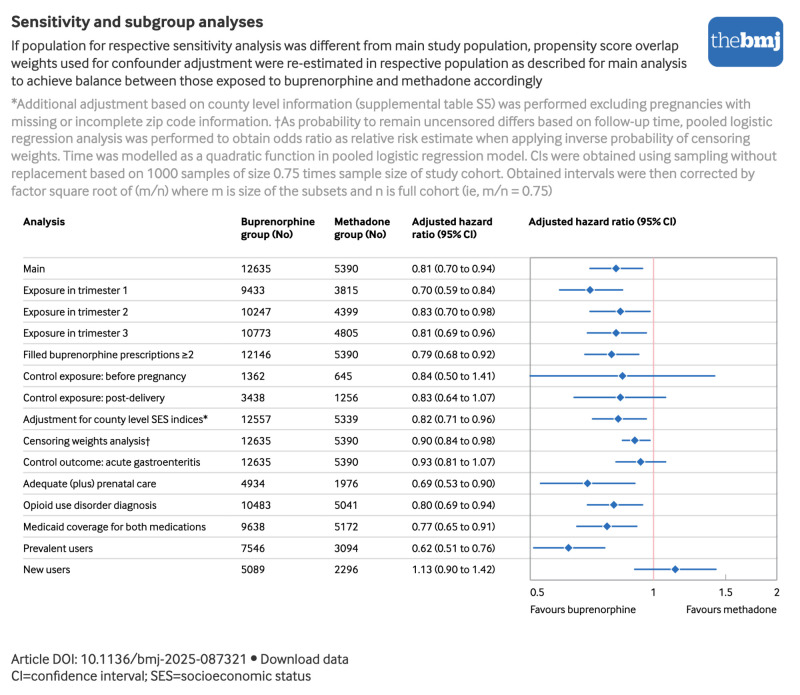
Adjusted relative risk of any neurodevelopmental disorder among children with prenatal exposure to buprenorphine versus methadone in main, sensitivity and subgroup analyses. An interactive version of this graphic is available at https://public.flourish.studio/visualisation/28044488/

Figure 4 and supplemental table S6 summarise the sensitivity and subgroup analyses. Restricting analyses to exposure within a specific trimester indicated similar results for the first, second, and third trimester. Results were also consistent for exposure to ≥2 dispensings of buprenorphine. Although estimates were relatively imprecise, results from the analyses using negative control exposure definitions were similar in direction and magnitude to those from the main analyses, indicating some potential for residual confounding. Adjustment for socioeconomic status did not change the results. When we applied censoring weights in addition to propensity score overlap weights, findings moved closer to the null, indicating some potential for informative censoring. Results for the negative control outcome of acute gastroenteritis did not suggest meaningful bias due to differences in healthcare seeking behaviour, although the risk was marginally lower among children with prenatal exposure to buprenorphine.

Results were consistent when restricting to pregnant individuals with an adequate level of prenatal care, a recorded diagnosis of opioid use disorder, and documented Medicaid coverage for both drugs. Most individuals initiated MOUD before pregnancy: 7546 (59.7%) in the buprenorphine group and 3094 (57.4%) in the methadone group. Findings differed substantially between prevalent and new use: with prevalent use, prenatal exposure to buprenorphine was associated with lower hazards of any neurodevelopmental disorder compared with prenatal exposure to methadone (adjusted hazard ratio 0.62, 0.51 to 0.76); this association was not observed with treatment initiation during pregnancy (1.13, 0.90 to 1.42).

Compared with prenatal exposure to methadone, the adjusted hazard ratio of any neurodevelopmental disorder for 2599 children with prenatal exposure to buprenorphine in combination with naloxone was 0.76 (0.60 to 0.96). In contrast, the adjusted hazard ratio for 4880 children with exposure to buprenorphine alone was 0.99 (0.81 to 1.20). Of those exposed to buprenorphine in combination with naloxone, treatment had been initiated before pregnancy in 68.9% versus only 26.9% of those exposed to buprenorphine alone. After stratification by prevalent and new use, the results for exposure to buprenorphine with naloxone and to buprenorphine without naloxone were both consistent with the analysis for buprenorphine overall (supplemental table S7). Both formulations were associated with lower hazards of neurodevelopmental disorders compared with methadone with prevalent use (adjusted hazard ratio 0.76 (0.55 to 1.06) for buprenorphine alone and 0.63 (0.47 to 0.84) for the combination treatment), but not with new use (1.15 (0.90 to 1.48) for buprenorphine alone and 1.07 (0.73 to 1.56) for the combination treatment).

## Discussion

In a nationwide cohort of 18 025 pregnant individuals with public (Medicaid) insurance, children with prenatal exposure to buprenorphine showed a similar to slightly lower risk of neurodevelopmental disorders compared with children with prenatal exposure to methadone, based on main, sensitivity, and subgroup analyses.

### Comparison with other studies

Few studies to date have directly compared neurodevelopmental outcomes among children with prenatal exposure to buprenorphine versus methadone. Reported results suggest either superior neurodevelopmental outcomes after prenatal exposure to buprenorphine^13^ ^14^ ^15^ ^16^ ^17^ or a similar risk between both drugs.^16^ ^17^ ^18^ ^19^ The available evidence is, however, limited to assessment of early neurodevelopmental outcomes,^13^ ^15^ ^17^ small sample sizes,^13^ ^14^ ^15^ ^16^ ^17^ ^18^ ^19^ and poor control of potential confounders.^16^ ^17^

Four studies with small sample sizes have provided findings on neurodevelopmental outcome beyond the first year of life. A Norwegian longitudinal study of 22 pregnancies exposed to methadone, nine exposed to buprenorphine, and 25 not exposed did not find any differences in attention problems reported on the Child Behavior Checklist between groups at a mean age of 52 months.^19^ Similarly, a study of 96 children from the Maternal Opioid Treatment: Human Experimental Research trial with up to 36 months of follow-up did not find evidence for a differential effect of buprenorphine exposure relative to methadone exposure on cognitive development, language abilities, sensory processing, or temperament.^18^ An observational cohort study among 247 infants found crude point estimates indicating a lower risk of developmental delays (risk ratio 0.61, 95% CI 0.27 to 1.36) and of behaviour, attention, or sleep problems (0.70, 0.25 to 1.94) with prenatal exposure to buprenorphine.^16^ A recent study limited to Medicaid data from Rhode Island found higher hazards of any neurodevelopmental disorders among 168 pregnancies exposed to methadone compared with 68 pregnancies exposed to buprenorphine (adjusted hazard ratio 2.07, 95% CI 1.14 to 3.74) and 167 unexposed pregnancies (2.51, 1.54 to 4.09).^14^ Results from all these studies are challenging to interpret, given the imprecision of the estimates due to small sample sizes and, in some instances, the potential for bias. Our study adds to this evidence by providing findings based on a large nationwide sample (12 635 pregnancies exposed to buprenorphine and 5390 exposed to methadone) with up to eight years of follow-up, comprehensive control of confounding, validated outcome definitions, and various sensitivity and subgroup analyses to carefully examine additional sources of bias.

### Synthesis of study findings

In the primary analysis, we observed slightly lower hazards of any neurodevelopmental disorder among children with prenatal exposure to buprenorphine versus methadone. Similar findings were obtained in sensitivity analyses accounting for potential exposure misclassification, potential differences in outcome diagnosis patterns due to differences in care setting, and residual confounding due to treatment indication, socioeconomic status, or regional and temporally varying treatment coverage by the insurance. Examination of acute gastroenteritis as a negative control outcome did not suggest meaningful surveillance bias.

When accounting for potential informative censoring using inverse probability of censoring weights, however, the relative risk attenuated. This suggests the observed lower hazards of neurodevelopmental disorders in the buprenorphine group may be partially explained by differences in follow-up and causes thereof, although it is challenging to conceptualise the potential underlying mechanisms behind such differential follow-up. Also, control exposure analyses examining maternal exposure before pregnancy or after delivery without exposure during pregnancy showed slightly lower hazards of any neurodevelopmental disorder with buprenorphine versus methadone. This suggests that potential residual confounding between the buprenorphine and methadone populations may account for the lower risk of neurodevelopmental disorders observed for buprenorphine. Finally, stratified analyses indicated a lower risk of any neurodevelopmental disorder from exposure to buprenorphine versus methadone with prevalent use, but not among those who initiated treatment during pregnancy. Potential causes underlying these findings remain unclear and require further investigation. It could be hypothesised that this observation may be related to factors such as differences in dosing regimens, treatment history, treatment retention, or risk of relapse. Overall, these data suggest that exposure to buprenorphine during pregnancy does not result in an increased risk of neurodevelopmental disorders compared with exposure to methadone during pregnancy. But the data also point to a lack of clear and compelling evidence that buprenorphine is associated with a substantially lower risk of neurodevelopmental disorders.

Although we obtained more imprecise estimates for some of the neurodevelopmental disorders when considered individually owing to smaller numbers, these analyses indicated that buprenorphine use during pregnancy compared with methadone use during pregnancy did not result in an increased risk of individual neurodevelopmental disorders such as attention deficit/hyperactivity disorder (adjusted hazard ratio 0.89, 95% CI 0.65 to 1.21) and autism spectrum disorder (0.74, 0.46 to 1.21). This suggests comparable long term safety of buprenorphine and methadone during pregnancy across a range of neurodevelopmental outcomes.

The observed crude cumulative incidences for the buprenorphine and methadone groups of, respectively, 33.9% and 33.1% for neurodevelopmental disorders overall, 20.5% and 17.4% for attention deficit/hyperactivity disorder, and 3.2% and 3.6% for autism spectrum disorder, as observed at age 8 years are higher than previously published at this age for the Medicaid population overall (any neurodevelopmental disorder: 23.9%, attention deficit/hyperactivity disorder: 14.5%, autism spectrum disorder: 1.6%).^30^ These results suggest this is a high risk population and emphasise the overall vulnerability of children with a history of maternal opioid use disorder.

### Strengths and limitations of this study

Most individuals with opioid use disorder in the US are insured through Medicaid. Utilising a large nationwide Medicaid cohort of pregnancies linked to the liveborn children ensured representativeness of the selected cohort and generalisability of our findings.^8^ Owing to the nature of health insurance claims data, there were no missing data for the study cohort. As recording of procedures and drug dispensations is directly tied to reimbursement, these data are expected to be complete. The lack of a recorded code for a specific medical condition within the respective assessment window was taken to mean the condition was not present. To minimise the possibility of under-ascertainment, we extended the covariate assessment window to the pregnancy period for chronic conditions that are not expected to be on the causal pathway. The outcome of interest in our study was neurodevelopmental disorders among liveborn children. As such, we conditioned on survival until the outcome could occur (ie, restriction to live births) and estimated the direct effect in the stratum of survivors. For the causal estimate to be valid, the assumption must be made that there is no uncontrolled common cause of pregnancy loss and neurodevelopmental disorders (ie, no collider bias), or that the treatment is not associated with pregnancy loss. Since there is no available evidence to indicate that buprenorphine versus methadone differentially affects pregnancy loss, we believe this to be a reasonable assumption. Even though a substantial proportion of children were lost to follow-up by 8 years of age, the absolute number of children under observation remained substantial given the large size of the birth cohort (fig 2 and supplemental figures S2 and S3). Most children were lost to follow-up because they reached the end of the study period rather than loss of insurance. Cohort studies with prospective data collection tend to focus on subtle manifestations of neurodevelopmental disorders using established developmental assessment batteries. Such information is not available in healthcare utilisation. While milder phenotypes of neurodevelopmental disorders or those with onset beyond age 8 years (eg, some learning difficulties) may be under-captured in this study, the large size of our cohort allowed us to evaluate the risk of more debilitating and clinically significant conditions (eg, autism spectrum disorder) assessed on the basis of validated algorithms.^23^ ^30^ As such, our study complements the information from smaller prospective studies. Another unique strength of our study was the triangulation approach to identify non-causal explanations of our findings by conducting a range of sensitivity analyses each addressing a different potential bias. This approach led to a cautious and conservative interpretation of the findings.

### Conclusion and implications

Treatment with opioid agonists during pregnancy is crucial to improve outcomes for both pregnant individuals and their children. Compared with methadone, buprenorphine treatment during pregnancy (alone or combined with naloxone) for opioid use disorder was not associated with an increased risk of any neurodevelopmental disorder. This conclusion coupled with previous findings of a lower risk of adverse neonatal outcomes should provide reassurance for providers and individuals considering treatment with buprenorphine during pregnancy.

**What is already known on this topic**

Buprenorphine and methadone are recommended for the treatment of opioid use disorder during pregnancy
These drugs differ in the way they are administered in clinical practice and in their pharmacology
Previous research has shown that buprenorphine is associated with a lower risk of adverse neonatal outcomes, but evidence on long term safety is lacking

**What this study adds**

In this US nationwide cohort study, the crude cumulative incidence of neurodevelopmental disorders at age 8 years was 34% among 12 635 children with prenatal exposure to buprenorphine and 33% among 5390 children with prenatal exposure to methadone
Results from the main, sensitivity, and subgroup analyses were consistent with no increased risk of long term adverse neurodevelopmental outcomes after prenatal exposure to buprenorphine versus methadone
These findings further support buprenorphine as a safe treatment option for opioid use disorder during pregnancy

## Data Availability

This study utilised US nationwide healthcare utilisation data from Medicaid, which may be obtained from the Centers for Medicare and Medicaid Services, but restrictions apply to the availability of these data owing to domestic laws and regulations.

## Web extra

Extra material supplied by authors

## Notes

Contributors: SF, KFH, LS, and BTB conceived and designed the study. SF, KFH, LS, SH-D, YZ, HM, and BTB were involved in data acquisition and establishment of the study database. SF and GH analysed the data. SF, KFH, LS, SH-D, YZ, GH, HEJ, HSC, JMD, KJG, BL, MT, and BTB were involved in data interpretation. SF, KFH, LS, and BTB drafted the manuscript. SH-D, YZ, GH, HM, HEJ, HSC, JMD, KJG, BL, and MT critically revised the manuscript for important intellectual content. SF, KFH, and BTB are the guarantors of the study. The corresponding author attests that all listed authors meet authorship criteria and that no others meeting the criteria have been omitted.

Transparency: The guarantors of the study affirm that this manuscript is an honest, accurate, and transparent account of the study being reported; that no important aspects of the study have been omitted; and that any discrepancies from the study as planned (and, if relevant, registered) have been explained.

Dissemination to participants and related patient and public communities: Key findings will be disseminated through academic presentations, and the authors will work with their public affairs team to promote visibility of this study in traditional and social media outlets.

Provenance and peer review: Not commissioned; externally peer reviewed.
