# Supplementary material for: Prenatal exposure to buprenorphine or methadone and adverse neurodevelopmental outcomes: population based cohort study
Source: BMJ. 2026 Apr 15;393:e087321. doi: 10.1136/bmj-2025-087321 (PMC13081224; doi:10.1136/bmj-2025-087321)
Supplement: Supplementary file 1 — Supplementary information: Supplemental tables S1-S7 and supplemental figures S1-S3 [file fris087321.ww.pdf]

<u>Supplementary material:</u> Prenatal Exposure to Buprenorphine or Methadone and Adverse Neurodevelopmental Outcomes

INDEX

Supplemental Table S1. Exposure definitions 2

Supplemental Table S2. Outcome definitions 3

Supplemental Table S3. Covariate definitions and assessment periods 5

Supplemental Table S4. Additional specifications for sensitivity and subgroup analyses 7

Supplemental Table S5. Covariates by exposure status 10

- A) Covariates included in main analysis 10
- B) County-level socioeconomic variables 14

Supplemental Table S6. Detailed results of sensitivity and subgroup analyses 16

Supplemental Table S7. Analyses stratified by buprenorphine alone and in combination with naloxone 18

Supplemental Figure S1. Study design diagram 19

Supplemental Figure S2. Cumulative incidence of any NDD by exposure status based on all available follow-up 20

Supplemental Figure S3. Cumulative incidence for individual neurodevelopmental disorders by exposure status 21

- A) Attention Deficit Hyperactivity Disorder (ADHD) 21
- B) Autism Spectrum Disorder (ASD) 22
- C) Behavioural Disorder 23
- D) Developmental Coordination Disorder 24
- E) Developmental Speech/Language Disorder 25
- F) Intellectual Disability 26
- G) Learning Difficulty 27

Supplemental Table S1. Exposure definitions

Exposure	Assessment window	Definition
Buprenorphine	Any exposure from LMP until the day prior to delivery and no exposure to methadone from 90 days prior to LMP until the day prior to delivery	Any of the following as specified below: Buprenorphine monoproduct or combination with naloxone
Buprenorphine monoproduct	Any exposure from LMP until the day prior to delivery and no exposure to methadone from 90 days prior to LMP until the day prior to delivery	Any of the following<br>≥ 1 dispensing of the following: buprenorphine hcl (tab subl., implant), buprenorphine (soler syr)<br>≥ 1 CPT/HCPCS: J0570 (on/after Jan 1, 2017), J0571 (on/after Jan 1, 2015)
Buprenorphine combination with naloxone	Any exposure from LMP until the day prior to delivery and no exposure to methadone from 90 days prior to LMP until the day prior to delivery	Any of the following<br>≥ 1 dispensing of buprenorphine hcl/naloxone hcl (any formulation)<br>≥ 1 CPT/HCPCS: J0573 (on/after Jan 1, 2017), J0572/J0574/J0575 (on/after Jan 1, 2016)
Methadone	Any exposure from LMP until the day prior to delivery and no exposure to buprenorphine from 90 days prior to LMP until the day prior to delivery	≥ 1 of CPT/HCPCS: H0020 or S0109

Abbreviations: CPT=Current Procedural Terminology, HCPCS=Healthcare Common Procedure Coding System, LMP = Last Menstrual Period

Supplemental Table S2. Outcome definitions[a]

Outcome	Assessment window[a,b]	Definition[a,c]	ICD-9 Dx[a, d]	ICD-10 Dx[a, e]	PPV (95% CI)[d]
Any Neurodevelopmental Disorder		Presence of any of the following as specified below: Autism Spectrum Disorder, Attention Deficit Hyperactivity Disorder, Learning Difficulty, Speech/Language Disorder, Intellectual disability, Developmental Coordination Disorder, Behavioural Disorder			
Autism Spectrum Disorder	At ≥ 1 year of age	≥ 2 dates with ICD Dx	299.xx (except 299.1x)	F84.x (except F84.2, F84.3)	0.94 (0.83-0.99)
Attention Deficit Hyperactivity Disorder	At ≥ 2 years of age	any of the following:<br>≥ 2 dates with ICD Dx;<br>≥ 2 dispensings of atomoxetine, clonidine, guanfacine, (dextro/lisdex)amphetamine, (dex)methylphenidate;<br>or ≥ 1 date with ICD Dx & ≥ 1 dispensing	314.xx	F90.x	0.88 (0.76-0.95)
Behavioural Disorder	At ≥ 2 years of age	≥ 2 dates with ICD Dx	312.xx, 313.xx	F63.xx, F91.x, F93.8, F93.9, F94.x, F98.8, F98.9	0.92 (0.81-0.98)
Developmental Coordination Disorder	Any age	≥ 2 dates with ICD Dx	315.4	F82	0.38 (0.25-0.53) [if including coordination issues: 0.90 (0.82-0.98)]
Developmental Speech/Language Disorder	At ≥ 1.5 years of age	≥ 2 dates with ICD Dx	315.3x (except 315.34)	F80.xx (except F80.4), H93.25	0.98 (0.89-1.00)
Learning Difficulty	At ≥ 2 years of age	≥ 1 date with ICD Dx	315.0x, 315.1, 315.2	F81.0, F81.2, F81.8x, R48.0	0.98 (0.89-1.00)
Intellectual Disability	At ≥ 2 years of age	≥ 2 dates with ICD Dx	317, 318.x, 319	F70-F79	0.82 (0.69-0.91)

Abbreviations: CI=confidence interval, Dx=Diagnosis, ICD=International Classification of Disease, PPV=positive predictive value

[a] Outcome definitions as specified in previously validated claims-based algorithms by our group. (Ref: Straub L, Bateman BT, Hernandez-Diaz S, et al. Validity of claims-based algorithms to identify neurodevelopmental disorders in children. Pharmacoepidemiol Drug Saf 2021;30(12):1635-42. doi: 10.1002/pds.5369)

[b] Assessment windows were chosen based on a minimum age where a correct diagnosis can be made based on diagnosis recommendations for the individual neurodevelopmental disorders. (Ref: Straub L, Bateman BT, Hernandez-Diaz S, et al. Validity of claims-based algorithms to identify neurodevelopmental disorders in children. Pharmacoepidemiol Drug Saf 2021;30(12):1635-42. doi: 10.1002/pds.5369)

[c] These algorithms were designed to maximize specificity by requiring ≥ 2 medical encounters with a specified diagnostic code (i.e., to reduce potential for coding errors and rule-out diagnoses). Learning difficulty was an exception from this requirement as multiple dates with respective billing codes were unlikely to be present due to learning difficulty generally not requiring services reimbursable through health insurance. ADHD medication are unlikely to be prescribed to children for conditions other than ADHD; thus, these were also utilised in the outcome algorithm. (Ref: Straub L, Bateman BT, Hernandez-Diaz S, et al. Validity of claims-based algorithms to identify neurodevelopmental disorders in children. Pharmacoepidemiol Drug Saf 2021;30(12):1635-42. doi: 10.1002/pds.5369)

[d] The outcome definitions based on ICD-9 codes were previously validated for each individual neurodevelopmental disorder by medical record review of a random sample of 50 cases identified using the specified claims-based algorithm. The medical record was reviewed by two physicians separately, assessing presence of the respective outcome based on meeting diagnostic criteria listed in the Diagnostic and Statistical Manual of Mental Disorders (DSM-5). Positive predictive values were then calculated based on the proportion of algorithm-derived cases confirmed through chart review. The positive predictive values stated here are as reported in the previously published validation study. For further information on exploration of false positive cases, please refer to the original paper. (Ref: Straub L, Bateman BT, Hernandez-Diaz S, et al. Validity of claims-based algorithms to identify neurodevelopmental disorders in children. Pharmacoepidemiol Drug Saf 2021;30(12):1635-42. doi: 10.1002/pds.5369)

[e] The ICD-9 definitions were translated to ICD-10 by: 1) applying a forward-backward mapping method created by the Centers for Medicare & Medicaid Services and the Centers for Disease Control and Prevention and review of the identified codes, 2) exploration of the ICD-10 data dictionary to identify additional codes of interest. (Ref: Straub L, Bateman BT, Hernandez-Diaz S, et al. Validity of claims-based algorithms to identify neurodevelopmental disorders in children. Pharmacoepidemiol Drug Saf 2021;30(12):1635-42. doi: 10.1002/pds.5369)

Supplemental Table S3. Covariate definitions and assessment periods

<table>
<tr><th>Category</th><th>Assessment window</th><th>Variables[a]</th></tr>
<tr><td>Personal characteristics</td><td>At delivery</td><td>Maternal age, maternal race/ethnicity, residential state/region, year of delivery</td></tr>
<tr><td>Conditions describing maternal substance dependence other than opioids</td><td rowspan="4">From 90 days prior to LMP until one day prior to delivery</td><td>Alcohol misuse, tobacco use, comorbid other (non-opioid) substance use disorders (including diagnosed misuse, dependence or poisoning with sedatives/hypnotics, cocaine, cannabis, hallucinogens, psychostimulants, or amphetamines)</td></tr>
<tr><td>Mental health conditions</td><td>Any psychotherapy, attention deficit hyperactivity disorder, anxiety disorder, bipolar disorder, depression, post-traumatic stress disorder, schizophrenia/schizoaffective disorder, sleep disorder, adjustment disorder, chronic fatigue syndrome, personality disorder, psychotic disorder, behavioural disorder,
other mental health disorder (including delirium, dementia, eating disorder, self-inflicted injury, somatoform spectrum disorder, tic disorder, autism spectrum disorder, developmental coordination disorder, developmental speech/language disorder, intellectual disability, and learning disorder)</td></tr>
<tr><td>Pain conditions</td><td>Chronic pain, abdominal pain, orthopaedic injury, arthritis, arthrosis or musculoskeletal pain, back and neck pain, dental, fibromyalgia, joint pain, migraine or other headache, neuropathic pain</td></tr>
<tr><td>Other comorbid conditions</td><td>Anaemia, pregestational hypertension, pregestational diabetes, nutritional deficiencies, obesity or overweight, renal disease, epilepsy, neuromuscular disorder, asthma, autoimmune disorder, influenza, infection with a TORCH syndrome relevant virus, hyperemesis,
Obstetric Comorbidity Index[b]</td></tr>
<tr><td>Proxies for severity of opioid use disorder</td><td rowspan="3">From 90 days prior to LMP until end of first half of pregnancy (140 days after LMP)</td><td>Number of opioid related emergency department visits, number of opioid related hospital admissions, any residential substance use disorder treatment</td></tr>
<tr><td>Medical conditions associated with opioid use disorder</td><td>Sexually transmitted disease, hepatitis B or C, HIV, bacteraemia, sepsis, endocarditis</td></tr>
<tr><td>Proxies for severity of mental health conditions</td><td>Number of distinct main mental health diagnoses, number of mental health-related emergency department visits, number of mental health-related hospital admissions</td></tr>
</table>

Category	Assessment window	Variables[a]
Healthcare utilisation	From 90 days prior to LMP until end of first half of pregnancy (140 days after LMP)	Number of unique diagnosis codes, number of prescribed generic drugs, number of emergency department visits, number of hospital admissions, number of outpatient visits
Concomitant medications		Antidepressant, antipsychotic, benzodiazepine, anxiolytic or other hypnotic, barbiturate, mood stabilizer or gabapentinoid, psychostimulant, non-steroidal anti-inflammatory drug, acetaminophen, antibiotic, corticosteroid, triptan, anti-viral agent, anti-nausea mediation, oral antidiabetic or insulin, antihypertensive, progestin, known teratogenic agents, suspected teratogenic agents; cumulative dose of prescription opioids
Multiple gestation pregnancy	From LMP until 60 days after delivery	
Adequacy of Prenatal Care Utilization Index[c]	From LMP until one day prior to delivery	Categories: Inadequate, intermediate, adequate, adequate plus

Abbreviations: LMP=Last Menstrual Period; TORCH=toxoplasmosis, other(syphilis, varicella-zoster, parvovirus B19), rubella, cytomegalovirus, herpes simplex; HIV=human immunodeficiency virus

[a] Specification of all covariate definitions and codes can be obtained from the authors upon request.

[b] Bateman BT, Mhyre JM, Hernandez-Diaz S, et al. Development of a comorbidity index for use in obstetric patients. Obstet Gynecol 2013;122(5):957-65. doi: 10.1097/AOG.0b013e3182a603bb

[c] Kotelchuck M. An evaluation of the Kessner Adequacy of Prenatal Care Index and a proposed Adequacy of Prenatal Care Utilization Index. Am J Public Health 1994;84(9):1414-20. doi: 10.2105/ajph.84.9.1414

Supplemental Table S4. Additional specifications for sensitivity and subgroup analyses

Sensitivity analysis	Modification compared with main analysis	Specification	Concern or motivation
Exposure in trimester 1	Exposure assessment window	Any exposure from **LMP until LMP+90 day**s and no exposure to comparator medication from 90 days prior to LMP until the day prior to delivery	Differential risk conditional on timing of exposure during pregnancy
Exposure in trimester 2	Exposure assessment window	Any exposure from **LMP+91 until LMP+180 days** and no exposure to comparator medication from 90 days prior to LMP until the day prior to delivery	
Exposure in trimester 3	Exposure assessment window	Any exposure from **LMP+181 days until delivery-1 day** and no exposure to comparator medication from 90 days prior to LMP until the day prior to delivery	
Filled BUP prescriptions ≥ 2	Definition for buprenorphine exposure	≥ 2 dispensings of buprenorphine formulations	Exposure misclassification, higher certainty of capturing actual buprenorphine consumption
Control exposure: prior to pregnancy	Exposure assessment window	Any (maternal) exposure from **365 days until one day prior to LMP**, no exposure to comparator medication during this period, no exposure to either medication from LMP to one day prior to delivery	Residual/unmeasured confounding, exclude biological pathway
Control exposure: initiation post-delivery	Exposure assessment window	Any (maternal) exposure from **delivery to 9 months after delivery**, no exposure to comparator medication during this period, no exposure to either medication from 90 days prior to LMP to one day prior to delivery	
Additional adjustment for county-level SES indices	Adjustment	Including measures of socio-economic status based on county-level information (unemployment rate, poverty rate, rate of education level below high school diploma, proximity to metropolitan area) in PS model in addition to all covariates specified for main analysis;<br>excluding those with missing information for these measures	Residual confounding due to differences in socio-economic status

Sensitivity analysis	Modification compared with main analysis	Specification	Concern or motivation
Censoring-weights analysis	Adjustment	(1) using pregnancy-months long data format,<br>(2) estimation of stabilised inverse-probability of censoring weights (IPCW) based on logistic regression model to estimate probability to remain uncensored at time t of follow-up in months, conditional on timing of initiation, using all covariates as described for overlap weights in statistical analysis section,<br>(3) combine IPCW and PS overlap weights through multiplication, truncate combined weights at 99th percentile,<br>(4) outcome model: pooled logistic regression analysis using combined weights modelling time as a quadratic function	Selection bias due to informative censoring
Control outcome: Acute gastroenteritis	Outcome definition	Acute gastroenteritis: ≥ 1 ICD-9 Dx 008.6x, 008.8, 009.xx; or ICD-10 Dx A08.x, A09; at any age	Surveillance bias leading to differences in outcome detection/recording
Adequate (plus) prenatal care	Inclusion/Exclusion	Restrict to pregnancies with adequate or adequate plus level of prenatal care as quantified by the Adequacy of Prenatal Care Utilization Index[a]	Differential outcome diagnosis pattern due to difference in care setting between compared groups
OUD diagnosis	Inclusion/Exclusion	Restrict to pregnancies with a recorded diagnosis of opioid use disorder between 90 days prior to pregnancy and the day prior to delivery	Residual confounding due to treatment indication
Medicaid coverage for both medications	Inclusion/Exclusion	Restrict to pregnancies with insurance coverage for both treatments by Medicaid in the respective state at timing of delivery	Residual confounding due to differences in insurance coverage of compared treatments by state and year
Prevalent use	Subgroup	Exposure initiation between 90 days to one day prior to LMP	Differences in motivation to initiate OAT and choice of medication due to awareness of pregnancy
New use	Subgroup	Exposure initiation between LMP and the day prior to delivery (**no** exposure from 90 days to one day prior to LMP)	

Sensitivity analysis	Modification compared with main analysis	Specification	Concern or motivation
Buprenorphine alone	Definition for buprenorphine exposure	Only exposed to "Buprenorphine monoproduct" as defined in supplemental table S1	Differential effect by the two buprenorphine treatment options
Buprenorphine + naloxone	Definition for buprenorphine exposure	Only exposed to "Buprenorphine combination with naloxone" as defined in supplemental table S1	

Abbreviations: BUP = buprenorphine, Dx=Diagnosis, ICD=International Classification of Disease, IPCW = inverse probability of censoring weights, LMP = last menstrual period, OAT = opioid agonist treatment, OUD = opioid use disorder, PS = propensity score.

[a] Kotelchuck M. An evaluation of the Kessner Adequacy of Prenatal Care Index and a proposed Adequacy of Prenatal Care Utilization Index. Am J Public Health 1994;84(9):1414-20. doi: 10.2105/ajph.84.9.1414

Supplemental Table S5. Covariates by exposure status

A) Covariates included in main analysis

	Full cohort			Weighted population	
	Buprenorphine	Methadone	Stand. Diff.[a]	Buprenorphine	Methadone
n	12635	5390		2665.62	2665.62
**DEMOGRPAHICS**					
**Age**, mean (SD)	28.26 (4.64)	28.47 (4.90)	-0.04	28.36 (4.77)	28.36 (4.72)
**Race/Ethnicity**					
White	11092 (87.8)	4055 (75.2)	**0.33**	2167.8 (81.3)	2167.8 (81.3)
Black/African American	352 (2.8)	493 (9.1)	**-0.27**	140.4 (5.3)	140.4 (5.3)
Hispanic/Latino	438 (3.5)	406 (7.5)	**-0.18**	156.7 (5.9)	156.7 (5.9)
Asian/Pacific Islander	24 (0.2)	36 (0.7)	-0.07	10.3 (0.4)	10.3 (0.4)
Unknown/Other	729 (5.8)	400 (7.4)	-0.07	190.4 (7.1)	190.4 (7.1)
**Region**					
Northeast	3955 (31.3)	1404 (26.0)	**0.12**	761.0 (28.5)	761.0 (28.5)
Midwest	4444 (35.2)	1433 (26.6)	**0.19**	793.1 (29.8)	793.1 (29.8)
South	3369 (26.7)	1442 (26.8)	<0.01	745.0 (27.9)	745.0 (27.9)
West	867 (6.9)	1111 (20.6)	**-0.41**	366.6 (13.8)	366.6 (13.8)
**Year of delivery[b]**					
2003	5 (0.0)	22 (0.4)	-0.08	2.9 (0.1)	2.9 (0.1)
2004	16 (0.1)	82 (1.5)	**-0.16**	11.2 (0.4)	11.2 (0.4)
2005	34 (0.3)	94 (1.7)	**-0.15**	18.7 (0.7)	18.7 (0.7)
2006	55 (0.4)	109 (2.0)	**-0.14**	29.8 (1.1)	29.8 (1.1)
2007	119 (0.9)	118 (2.2)	**-0.10**	41.2 (1.5)	41.2 (1.5)
2008	208 (1.6)	134 (2.5)	-0.06	60.1 (2.3)	60.1 (2.3)
2009	403 (3.2)	167 (3.1)	0.01	88.8 (3.3)	88.8 (3.3)
2010	626 (5.0)	290 (5.4)	-0.02	140.8 (5.3)	140.8 (5.3)
2011	884 (7.0)	337 (6.3)	0.03	173.5 (6.5)	173.5 (6.5)
2012	1010 (8.0)	365 (6.8)	0.05	202.2 (7.6)	202.2 (7.6)
2013	1415 (11.2)	491 (9.1)	0.07	274.3 (10.3)	274.3 (10.3)
2014	1384 (11.0)	612 (11.4)	-0.01	312.0 (11.7)	312.0 (11.7)
2015	1198 (9.5)	445 (8.3)	0.04	248.5 (9.3)	248.5 (9.3)
2016	1626 (12.9)	679 (12.6)	0.01	332.7 (12.5)	332.7 (12.5)
2017	2095 (16.6)	853 (15.8)	0.02	427.0 (16.0)	427.0 (16.0)
2018	1557 (12.3)	592 (11.0)	0.04	301.9 (11.3)	301.9 (11.3)
**Multiple gestation pregnancy**	261 (2.1)	89 (1.7)	0.03	47.5 (1.8)	47.5 (1.8)
**MATERNAL SUBSTANCE DEPENDENCE, other than opioids**					
**Alcohol misuse**	959 (7.6)	249 (4.6)	**0.12**	151.3 (5.7)	151.3 (5.7)
**Tobacco use**	6156 (48.7)	2160 (40.1)	**0.18**	1189.3 (44.6)	1189.3 (44.6)
**Comorbid other (non-opioid) substance use disorders**	2510 (19.9)	860 (16.0)	**0.10**	468.3 (17.6)	468.3 (17.6)
**Residential substance use disorder treatment**	190 (1.5)	38 (0.7)	0.08	25.9 (1.0)	25.9 (1.0)

	Full cohort			Weighted population	
	Buprenorphine	Methadone	Stand. Diff.[a]	Buprenorphine	Methadone
**CONDITIONS ASSOCIATED WITH OUD**					
**Bacteraemia, Sepsis or Endocarditis**	103 (0.8)	45 (0.8)	<0.01	24.9 (0.9)	24.9 (0.9)
**Sexually transmitted disease, hepatitis B/C or HIV**	1596 (12.6)	738 (13.7)	-0.03	364.7 (13.7)	364.7 (13.7)
**INDICATORS FOR OUD SEVERITY**					
**Number of opioid related emergency department visits**					
0	11893 (94.1)	4982 (92.4)	0.07	2468.8 (92.6)	2468.8 (92.6)
1	598 (4.7)	313 (5.8)	-0.05	152.9 (5.7)	152.9 (5.7)
≥2	144 (1.1)	95 (1.8)	-0.05	43.9 (1.6)	43.9 (1.6)
**Number of opioid related hospital admissions**					
0	11746 (93.0)	5058 (93.8)	-0.04	2482.1 (93.1)	2482.1 (93.1)
1	731 (5.8)	274 (5.1)	0.03	149.4 (5.6)	149.4 (5.6)
≥2	158 (1.3)	58 (1.1)	0.02	34.1 (1.3)	34.1 (1.3)
**COMORBIDITY BURDEN**					
**Anaemia**	1143 (9.0)	473 (8.8)	0.01	242.3 (9.1)	242.3 (9.1)
**Hypertension, pregestational**	1008 (8.0)	466 (8.6)	-0.02	222.4 (8.3)	222.4 (8.3)
**Diabetes, pregestational**	308 (2.4)	125 (2.3)	0.01	59.9 (2.2)	59.9 (2.2)
**Nutritional deficiencies**	368 (2.9)	109 (2.0)	0.06	62.4 (2.3)	62.4 (2.3)
**Obesity or overweight**	992 (7.9)	540 (10.0)	-0.08	239.6 (9.0)	239.6 (9.0)
**Renal disease**	82 (0.6)	31 (0.6)	0.01	14.8 (0.6)	14.8 (0.6)
**Epilepsy**	508 (4.0)	202 (3.7)	0.01	105.1 (3.9)	105.1 (3.9)
**Neuromuscular disorder**	140 (1.1)	41 (0.8)	0.04	22.9 (0.9)	22.9 (0.9)
**Asthma**	1162 (9.2)	610 (11.3)	-0.07	279.6 (10.5)	279.6 (10.5)
**Autoimmune disorder**	243 (1.9)	93 (1.7)	0.02	44.3 (1.7)	44.3 (1.7)
**Influenza**	148 (1.2)	50 (0.9)	0.02	26.5 (1.0)	26.5 (1.0)
**Infection with a TORCH syndrome relevant virus**	545 (4.3)	223 (4.1)	0.01	110.4 (4.1)	110.4 (4.1)
**Hyperemesis**	3258 (25.8)	1432 (26.6)	-0.02	700.7 (26.3)	700.7 (26.3)
**Obstetric Comorbidity Index[c]**					
0-1	703 (5.6)	222 (4.1)	0.07	77.6 (2.9)	77.6 (2.9)
2	5152 (40.8)	2161 (40.1)	0.01	1109.0 (41.6)	1109.0 (41.6)
≥ 3	6780 (53.7)	3007 (55.8)	-0.04	1479.0 (55.5)	1479.0 (55.5)
**MENTAL HEALTH CONDITIONS**					
**ADHD**	752 (6.0)	247 (4.6)	0.06	147.7 (5.5)	147.7 (5.5)
**Anxiety disorder**	3928 (31.1)	1259 (23.4)	**0.17**	722.3 (27.1)	722.3 (27.1)
**Bipolar disorder**	1486 (11.8)	557 (10.3)	0.05	309.5 (11.6)	309.5 (11.6)
**Depression**	4015 (31.8)	1281 (23.8)	**0.18**	737.3 (27.7)	737.3 (27.7)
**Post-Traumatic Stress Disorder**	862 (6.8)	388 (7.2)	-0.02	199.4 (7.5)	199.4 (7.5)
**Schizophrenia/Schizo-affective disorder**	54 (0.4)	28 (0.5)	-0.01	12.3 (0.5)	12.3 (0.5)
**Sleep disorder**	617 (4.9)	188 (3.5)	0.07	105.0 (3.9)	105.0 (3.9)

	Full cohort			Weighted population	
	Buprenorphine	Methadone	Stand. Diff.[a]	Buprenorphine	Methadone
**Adjustment disorder**	197 (1.6)	67 (1.2)	0.03	31.5 (1.2)	31.5 (1.2)
**Chronic fatigue syndrome**	820 (6.5)	278 (5.2)	0.06	150.0 (5.6)	150.0 (5.6)
**Personality disorder**	177 (1.4)	76 (1.4)	<0.01	39.1 (1.5)	39.1 (1.5)
**Psychotic disorder**	151 (1.2)	69 (1.3)	-0.01	36.0 (1.4)	36.0 (1.4)
**Behavioural disorder**	113 (0.9)	44 (0.8)	0.01	22.3 (0.8)	22.3 (0.8)
**Other MH disorder[d]**	5505 (43.6)	1863 (34.6)	**0.19**	1042.2 (39.1)	1042.2 (39.1)
**SEVERITY OF MENTAL HEALTH BURDEN**					
**Number of distinct main MH diagnoses**					
0	1918 (15.2)	600 (11.1)	**0.12**	287.5 (10.8)	287.5 (10.8)
1 or 2	5359 (42.4)	3068 (56.9)	**-0.29**	1407.1 (52.8)	1407.1 (52.8)
≥ 3	5358 (42.4)	1722 (31.9)	**0.22**	971.0 (36.4)	971.0 (36.4)
**Number of MH-related emergency department visits**					
0	10024 (79.3)	4320 (80.1)	-0.02	2091.7 (78.5)	2091.7 (78.5)
1	1745 (13.8)	702 (13.0)	0.02	378.6 (14.2)	378.6 (14.2)
≥2	866 (6.9)	368 (6.8)	<0.01	195.4 (7.3)	195.4 (7.3)
**Number of MH-related hospital admissions**					
0	11511 (91.1)	4955 (91.9)	-0.03	2429.4 (91.1)	2429.4 (91.1)
1	893 (7.1)	336 (6.2)	0.03	182.8 (6.9)	182.8 (6.9)
≥2	231 (1.8)	99 (1.8)	<0.01	53.4 (2.0)	53.4 (2.0)
**Psychotherapy**	2602 (20.6)	1131 (21.0)	-0.01	575.7 (21.6)	575.7 (21.6)
**PAIN CONDITIONS**					
**Chronic pain**	1986 (15.7)	751 (13.9)	0.05	408.4 (15.3)	408.4 (15.3)
**Abdominal pain**	4406 (34.9)	2002 (37.1)	-0.05	978.4 (36.7)	978.4 (36.7)
**Orthopaedic injury**	1344 (10.6)	564 (10.5)	0.01	294.0 (11.0)	294.0 (11.0)
**Arthritis, arthrosis or musculoskeletal pain**	2301 (18.2)	932 (17.3)	0.02	482.8 (18.1)	482.8 (18.1)
**Back and neck pain**	3015 (23.9)	1103 (20.5)	0.08	591.0 (22.2)	591.0 (22.2)
**Dental**	1706 (13.5)	788 (14.6)	-0.03	390.9 (14.7)	390.9 (14.7)
**Fibromyalgia**	369 (2.9)	134 (2.5)	0.03	75.2 (2.8)	75.2 (2.8)
**Joint pain**	1235 (9.8)	476 (8.8)	0.03	259.5 (9.7)	259.5 (9.7)
**Migraineor or other headache**	627 (5.0)	233 (4.3)	0.03	120.5 (4.5)	120.5 (4.5)
**Neuropathic pain**	932 (7.4)	359 (6.7)	0.03	198.2 (7.4)	198.2 (7.4)
**CONCOMITTANT MEDICATIONS**					
**Antidepressants**	4986 (39.5)	1486 (27.6)	**0.25**	858.8 (32.2)	858.8 (32.2)
**Antipsychotics**	1371 (10.9)	465 (8.6)	0.08	254.2 (9.5)	254.2 (9.5)
**Benzodiazepines**	2417 (19.1)	936 (17.4)	0.05	491.9 (18.5)	491.9 (18.5)
**Other anxiolytics or hypnotics**	2469 (19.5)	742 (13.8)	**0.16**	426.6 (16.0)	426.6 (16.0)
**Barbiturates**	380 (3.0)	100 (1.9)	0.08	60.4 (2.3)	60.4 (2.3)
**Mood stabilizers or gabapentinoids**	2248 (17.8)	682 (12.7)	**0.14**	400.0 (15.0)	400.0 (15.0)
**Psychostimulants**	973 (7.7)	257 (4.8)	**0.12**	165.1 (6.2)	165.1 (6.2)
**Non-steroidal anti-inflammatory drugs**	3160 (25.0)	1218 (22.6)	0.06	626.0 (23.5)	626.0 (23.5)
**Acetaminophen**	3440 (27.2)	1704 (31.6)	**-0.10**	817.0 (30.6)	817.0 (30.6)

	Full cohort			Weighted population	
	**Buprenorphine**	**Methadone**	**Stand. Diff.[a]**	**Buprenorphine**	**Methadone**
**Antibiotics**	7163 (56.7)	2693 (50.0)	**0.14**	1418.7 (53.2)	1418.7 (53.2)
**Corticosteroids**	2005 (15.9)	784 (14.5)	**0.04**	399.1 (15.0)	399.1 (15.0)
**Triptans**	368 (2.9)	116 (2.2)	**0.05**	61.2 (2.3)	61.2 (2.3)
**Anti-viral agents**	660 (5.2)	231 (4.3)	0.04	123.8 (4.6)	123.8 (4.6)
**Anti-nausea mediations**	5230 (41.4)	1868 (34.7)	**0.14**	999.2 (37.5)	999.2 (37.5)
**Oral antidiabetics or insulin**	121 (1.0)	55 (1.0)	0.01	24.4 (0.9)	24.4 (0.9)
**Antihypertensives**	1458 (11.5)	414 (7.7)	**0.13**	240.6 (9.0)	240.6 (9.0)
**Progestins**	710 (5.6)	265 (4.9)	0.03	138.7 (5.2)	138.7 (5.2)
**Known teratogenics**	50 (0.4)	19 (0.4)	0.01	9.5 (0.4)	9.5 (0.4)
**Other suspected teratogenics**	2125 (16.8)	741 (13.7)	0.09	401.7 (15.1)	401.7 (15.1)
**Cumulative dose of prescription opioids, oral morphine mg equivalent**					
0	9117 (72.2)	3612 (67.0)	**0.11**	1804.0 (67.7)	1804.0 (67.7)
0.1-150	1338 (10.6)	657 (12.2)	-0.05	316.9 (11.9)	316.9 (11.9)
150-640	1012 (8.0)	524 (9.7)	-0.06	246.5 (9.2)	246.5 (9.2)
> 640	1168 (9.2)	597 (11.1)	-0.06	298.3 (11.2)	298.3 (11.2)
**HEALTHCARE UTILISATION**					
**Number of unique diagnosis codes**					
0-7	2609 (20.6)	1334 (24.7)	**-0.10**	603.8 (22.7)	603.8 (22.7)
8-11	2976 (23.6)	1295 (24.0)	-0.01	632.6 (23.7)	632.6 (23.7)
12-16	3346 (26.5)	1364 (25.3)	0.03	681.5 (25.6)	681.5 (25.6)
≥ 17	3704 (29.3)	1397 (25.9)	0.08	747.7 (28.1)	747.7 (28.1)
**Number of prescribed generic drugs**					
0	695 (5.5)	575 (10.7)	**-0.19**	219.1 (8.2)	219.1 (8.2)
1-3	3052 (24.2)	1499 (27.8)	-0.08	702.4 (26.3)	702.4 (26.3)
4-7	4346 (34.4)	1725 (32.0)	0.05	877.6 (32.9)	877.6 (32.9)
≥ 8	4542 (35.9)	1591 (29.5)	**0.14**	866.5 (32.5)	866.5 (32.5)
**Number of emergency department visits**					
0	4809 (38.1)	1907 (35.4)	0.06	937.9 (35.2)	937.9 (35.2)
1	2943 (23.3)	1246 (23.1)	<0.01	614.6 (23.1)	614.6 (23.1)
≥ 2	4883 (38.6)	2237 (41.5)	-0.06	1113.1 (41.8)	1113.1 (41.8)
**Number of hospital admissions**					
0	11308 (89.5)	4863 (90.2)	-0.02	2383.4 (89.4)	2383.4 (89.4)
1	1107 (8.8)	425 (7.9)	0.03	229.3 (8.6)	229.3 (8.6)
≥ 2	220 (1.7)	102 (1.9)	-0.01	52.9 (2.0)	52.9 (2.0)
**Number of outpatient visits**					
0-1	2228 (17.6)	2063 (38.3)	**-0.47**	677.5 (25.4)	677.5 (25.4)
2-4	2986 (23.6)	1132 (21.0)	0.06	668.0 (25.1)	668.0 (25.1)
5-10	3898 (30.9)	1122 (20.8)	**0.23**	701.8 (26.3)	701.8 (26.3)
≥ 11	3523 (27.9)	1073 (19.9)	**0.19**	618.3 (23.2)	618.3 (23.2)
**Category of Adequacy of Prenatal Care Utilization Index[e]**					
Inadequate	5614 (44.4)	2504 (46.5)	-0.04	1244.0 (46.7)	1244.0 (46.7)
Intermediate	2087 (16.5)	910 (16.9)	-0.01	442.1 (16.6)	442.1 (16.6)
Adequate	1967 (15.6)	741 (13.7)	0.05	379.0 (14.2)	379.0 (14.2)
Adequate Plus	2967 (23.5)	1235 (22.9)	0.01	600.4 (22.5)	600.4 (22.5)

	Full cohort			Weighted population	
	Buprenorphine	Methadone	Stand. Diff.[a]	Buprenorphine	Methadone
**Timing of buprenorphine/methadone initiation**					
Prior to pregnancy, within 90 days	7546 (59.7)	3094 (57.4)	0.05	1436.5 (53.9)	1436.5 (53.9)
Trimester 1	2167 (17.2)	801 (14.9)	0.06	462.6 (17.4)	462.6 (17.4)
Trimester 2	1815 (14.4)	844 (15.7)	-0.04	459.8 (17.2)	459.8 (17.2)
Trimester 3	1107 (8.8)	651 (12.1)	**-0.11**	306.7 (11.5)	306.7 (11.5)

B) County-level socioeconomic variables[f]

	Full cohort			Weighted population		
	Buprenorphine	Methadone	Stand. Diff.[a]	Buprenorphine	Methadone	Stand. Diff.[a]
n	12635	5390		2665.62	2665.62	
**Unemployment rate, quartile**						
Low	3605 (28.5)	1807 (33.5)	**-0.12**	755.0 (28.3)	933.2 (35.0)	**-0.15**
Low to moderate	3214 (25.4)	1329 (24.7)	0.04	663.4 (24.9)	700.9 (26.3)	-0.06
Moderate to high	2960 (23.4)	1169 (21.7)	0.06	626.8 (23.5)	563.4 (21.1)	0.07
High	2778 (22.0)	1034 (19.2)	0.08	602.5 (22.6)	439.0 (16.5)	**0.16**
**Poverty rate, quartile**						
Low	3497 (27.7)	1973 (36.6)	**-0.20**	742.9 (27.9)	1004.5 (37.7)	**-0.22**
Low to moderate	2832 (22.4)	1345 (25.0)	-0.07	577.0 (21.6)	675.3 (25.3)	**-0.10**
Moderate to high	3341 (26.4)	1132 (21.0)	**0.13**	717.0 (26.9)	566.9 (21.3)	**0.14**
High	2887 (22.8)	889 (16.5)	**0.16**	610.8 (22.9)	390.0 (14.6)	**0.22**
**Percentage of county with ≤ High School Diploma, quartile**						
Low	2642 (20.9)	1944 (36.1)	**-0.35**	634.6 (23.8)	896.4 (33.6)	**-0.23**
Low to moderate	2865 (22.7)	1454 (27.0)	**-0.11**	572.4 (21.5)	782.4 (29.4)	**-0.19**
Moderate to high	2310 (18.3)	1078 (20.0)	-0.06	493.7 (18.5)	544.1 (20.4)	-0.07
High	4740 (37.5)	863 (16.0)	**0.50**	947.0 (35.5)	413.7 (15.5)	**0.47**
**Proximity to metropolitan area**						
Metropolitan	9969 (78.9)	5007 (92.9)	**-0.43**	2095.0 (78.6)	2468.3 (92.6)	**-0.43**
Non-metropolitan	2108 (16.7)	281 (5.2)	**0.38**	450.6 (16.9)	144.7 (5.4)	**0.37**
Rural	480 (3.8)	51 (0.9)	**0.19**	102.0 (3.8)	23.6 (0.9)	**0.20**

Abbreviations: ADHD = Attention-Deficit Hyperactivity Disorder, HIV = human immunodeficiency virus, MH = Mental Health, OUD = Opioid use disorder, Stand. Diff. = Standardised difference

Displayed are frequency (%) if not specified otherwise. For assessment windows of different covariates, please see supplemental figure S1 and supplemental table S3.

[a] Standardised differences are provided as a measure of covariate balance between buprenorphine- and methadone-exposed pregnancies (Austin PC, Stuart EA. Moving towards best practice when using inverse probability of treatment weighting (IPTW) using the propensity score to estimate causal treatment effects in observational studies. Stat Med. 2015 Dec 10;34(28):3661-79. doi: 10.1002/sim.6607. Epub 2015 Aug 3. PMID: 26238958; PMCID: PMC4626409).
By definition, covariates included in the propensity score are perfectly balanced after overlap weighting. For these, standardised differences are not provided.

[b] After applying in- and exclusion criteria, no pregnancies with delivery from 2000-2002 were observed.

[c] Bateman BT, Mhyre JM, Hernandez-Diaz S, et al. Development of a comorbidity index for use in obstetric patients. Obstet Gynecol 2013;122(5):957-65. doi: 10.1097/AOG.0b013e3182a603bb

[d] Includes delirium, dementia, eating disorder, self-inflicted injury, somatoform spectrum disorder, tic disorder, autism spectrum disorder, developmental coordination disorder, developmental speech/language disorder, intellectual disability, and learning disorder.

[e] Kotelchuck M. An evaluation of the Kessner Adequacy of Prenatal Care Index and a proposed Adequacy of Prenatal Care Utilization Index. Am J Public Health 1994;84(9):1414-20. doi: 10.2105/ajph.84.9.1414

[f] These variables are based on zip code information which was missing or incomplete for 129 pregnancies. They were not included as covariates in the propensity score for the main analysis. Instead a sensitivity analysis with additional adjustment for these variables excluding records with missing information was performed. Zip code was assessed at time of last menstrual period and the socio-economic status (SES) measures were derived as follows: the Medicaid data was merged with county-level data on these SES measures provided by the US Department of Agriculture, after linkage of maternal zip codes with corresponding Federal Information Processing System county codes using crosswalk files provided by the US Department of Housing and Urban Development.

Supplemental Table S6. Detailed results of sensitivity and subgroup analyses

Analysis	Adjustment	Number of children at risk		Hazard Ratio (95% CI)	Cumulative incidence (95% CI)	
		Buprenorphine population	Methadone population		Buprenorphine population	Methadone population
Main	crude	12635	5390	0.94 (0.83-1.07)	34% (30-38%)	33% (29-37%)
	OWT			0.81 (0.70-0.94)	32% (27-36%)	36% (30-41%)
Exposure in trimester 1	crude	9433	3815	0.85 (0.73-0.98)	34% (29-39%)	34% (29-39%)
	OWT			0.70 (0.59-0.84)	30% (25-36%)	37% (30-43%)
Exposure in trimester 2	crude	10247	4399	0.92 (0.80-1.06)	34% (29-39%)	32% (28-37%)
	OWT			0.83 (0.70-0.98)	32% (26-37%)	34% (28-39%)
Exposure in trimester 3	crude	10773	4805	0.92 (0.81-1.06)	33% (29-38%)	33% (29-37%)
	OWT			0.81 (0.69-0.96)	31% (26-36%)	35% (30-41%)
Filled BUP prescriptions ≥ 2	crude	12146	5390	0.93 (0.82-1.05)	34% (30-38%)	33% (29-37%)
	OWT			0.79 (0.68-0.92)	32% (27-36%)	36% (30-41%)
Control exposure: prior to pregnancy	crude	1362	645	1.09 (0.73-1.64)	25% (18-32%)	30% (17-41%)
	OWT			0.84 (0.50-1.41)	21% (13-28%)	29% (15-41%)
Control exposure: initiation post-delivery	crude	3438	1256	0.78 (0.63-0.98)	32% (27-37%)	38% (31-44%)
	OWT			0.83 (0.64-1.07)	32% (25-37%)	37% (29-44%)
Additional adjustment for county-level SES indices	crude	12557	5339	0.94 (0.83-1.06)	34% (30-38%)	33% (29-37%)
	OWT			0.82 (0.71-0.96)	32% (27-37%)	37% (31-42%)
Censoring-weights analysis	IPCW*OWT	12635	5390	0.90 (0.84-0.98)[a]	51% (47-55%)[a]	55% (51-58%)[a]
Control outcome: Acute gastroenteritis	crude	12635	5390	0.94 (0.84-1.05)	16% (14-18%)	19% (16-21%)
	OWT			0.93 (0.81-1.07)	15% (13-17%)	18% (16-21%)
Adequate (plus) prenatal care	crude	4934	1976	0.92 (0.74-1.13)	31% (24-37%)	34% (26-41%)
	OWT			0.69 (0.53-0.90)	26% (20-32%)	38% (26-47%)
OUD diagnosis	crude	10483	5041	0.95 (0.83-1.09)	34% (29-39%)	34% (30-38%)
	OWT			0.80 (0.69-0.94)	31% (26-36%)	37% (31-42%)

Analysis	Adjustment	Number of children at risk		Hazard Ratio (95% CI)	Cumulative incidence (95% CI)	
		Buprenorphine population	Methadone population		Buprenorphine population	Methadone population
Medicaid coverage for both medications	crude	9638	5172	0.98 (0.85-1.12)	33% (28-37%)	33% (29-37%)
	OWT			0.77 (0.65-0.91)	30% (25-35%)	36% (31-42%)
Prevalent use	crude	7546	3094	0.79 (0.67-0.93)	33% (28-38%)	34% (28-39%)
	OWT			0.62 (0.51-0.76)	28% (23-33%)	37% (29-43%)
New use	crude	5089	2296	1.21 (0.99-1.48)	35% (29-41%)	32% (26-38%)
	OWT			1.13 (0.90-1.42)	36% (28-44%)	35% (26-42%)

Abbreviations: BUP=Buprenorphine, CI=confidence interval, IPCW=inverse probability of censoring weights, OUD=opioid use disorder, OWT= overlap weights, SES=socioeconomic status

If the population for the respective sensitivity analysis was different from the main study population, overlap weights were re-estimated in the respective population as described for the main analysis to achieve balance between the Buprenorphine and Methadone population accordingly.

[a] Pooled logistic regression with underlying pregnancy-month data was used to obtain an odds ratio as relative risk estimate when applying inverse-probability of censoring weights. Time was modelled as a quadratic function in the pooled logistic regression model. The hazard ratio obtained from a Cox regression model and odds ratio obtained from a pooled logistic regression model numerically identical. Interpretation of the odds ratio of the censoring-weights analysis corresponds to the interpretation of the hazard ratio of the other analyses. Confidence intervals were obtained using sampling without replacement based on 1000 samples of size 0.75 times the sample size of the study cohort. Obtained intervals were then corrected by factor squareroot of (m/n) where m is the size of the subsets and n is the full cohort (i.e., m/n = 0.75).

Supplemental Table S7. Analyses stratified by buprenorphine alone and in combination with naloxone

Analysis	Adjustment	Number at risk		Hazard Ratio (95% CI)	Cumulative incidence (95% CI)	
		Buprenorphine population	Methadone population		Buprenorphine population	Methadone population
**Overall**						
Main (any BUP vs. MET)	crude	12635	5390	0.94 (0.83-1.07)	34% (30-38%)	33% (29-37%)
	adjusted			0.81 (0.70-0.94)	32% (27-36%)	36% (30-41%)
BUP alone vs. MET	crude	4880	5390	1.10 (0.94-1.30)	32% (25-38%)	33% (29-37%)
	adjusted			0.99 (0.81-1.20)	33% (25-40%)	38% (30-44%)
BUP+NAL vs. MET	crude	2599	5390	0.78 (0.64-0.94)	35% (26-43%)	33% (29-37%)
	adjusted			0.76 (0.60-0.96)	36% (26-45%)	35% (29-40%)
**Prevalent use**						
any BUP vs. MET	crude	7546	3094	0.79 (0.67-0.93)	33% (28-38%)	34% (28-39%)
	adjusted			0.62 (0.51-0.76)	28% (23-33%)	37% (29-43%)
BUP alone vs. MET	crude	1315	3094	0.92 (0.70-1.20)	38% (20-52%)	34% (28-39%)
	adjusted			0.76 (0.55-1.06)	35% (20-47%)	39% (28-49%)
BUP+NAL vs. MET	crude	1792	3094	0.67 (0.53-0.85)	35% (22-45%)	34% (28-39%)
	adjusted			0.63 (0.47-0.84)	33% (20-44%)	37% (28-44%)
**New use**						
any BUP vs. MET	crude	5089	2296	1.21 (0.99-1.48)	35% (29-41%)	32% (26-38%)
	adjusted			1.13 (0.90-1.42)	36% (28-44%)	35% (26-42%)
BUP alone vs. MET	crude	3565	2296	1.25 (1.01-1.55)	30% (23-37%)	32% (26-38%)
	adjusted			1.15 (0.90-1.48)	32% (22-41%)	37% (27-45%)
BUP+NAL vs. MET	crude	807	2296	1.06 (0.76-1.48)	36% (22-48%)	32% (26-38%)
	adjusted			1.07 (0.73-1.56)	41% (22-56%)	31% (23-38%)

Abbreviations: BUP=buprenorphine, CI=confidence interval, MET=methadone, NAL=naloxone

Pregnancies exposed to both, buprenorphine alone and the combination of buprenorphine and naloxone, during pregnancy are only considered in the "any BUP vs. MET" analysis.
If the population for the respective analysis was different from the main study population, overlap weights for confounding adjustment were re-estimated in the respective population as described for the main analysis to achieve balance between the Buprenorphine and Methadone population accordingly.

Supplemental Figure S1. Study design diagram

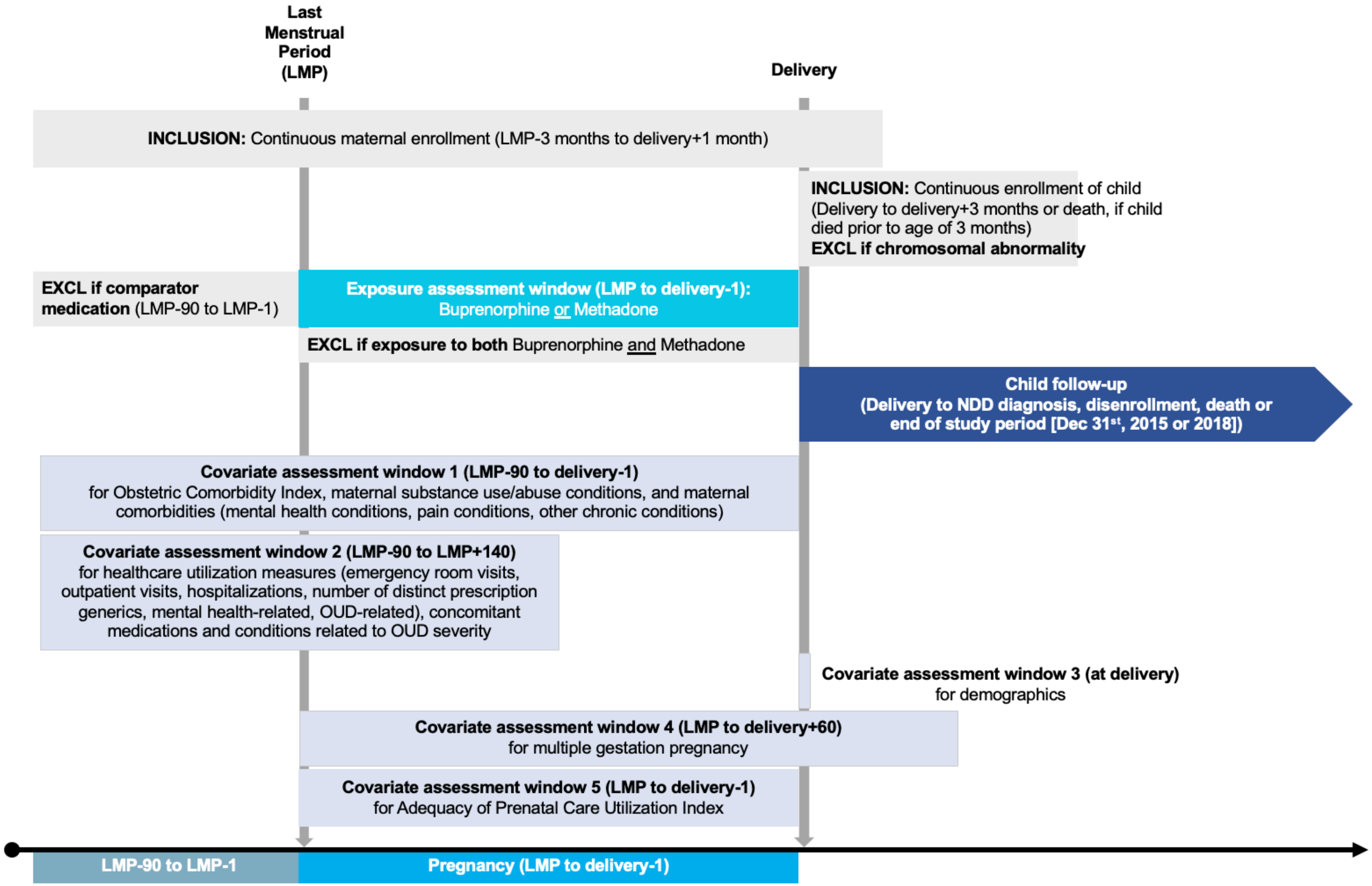

Abbreviations: EXCL = exclude, LMP = last menstrual period, NDD = Neurodevelopmental disorder, OUD = Opioid use disorder.
Number after specific time points (+/-) represent count in days if not specified otherwise. E.g. "delivery-1" denotes delivery minus one day (or the day before delivery). For details, please refer to supplemental table S1 and supplemental table S3.
End of study period was December 31st 2015 for children born in one of the following US states: CO, GA, IL, KY, MD, NH, NY, PA, PR, SC, TN, TX, UT, and December 31st, 2018 for children born in all other states.

Supplemental Figure S2. Cumulative incidence of any NDD by exposure status based on all available follow-up

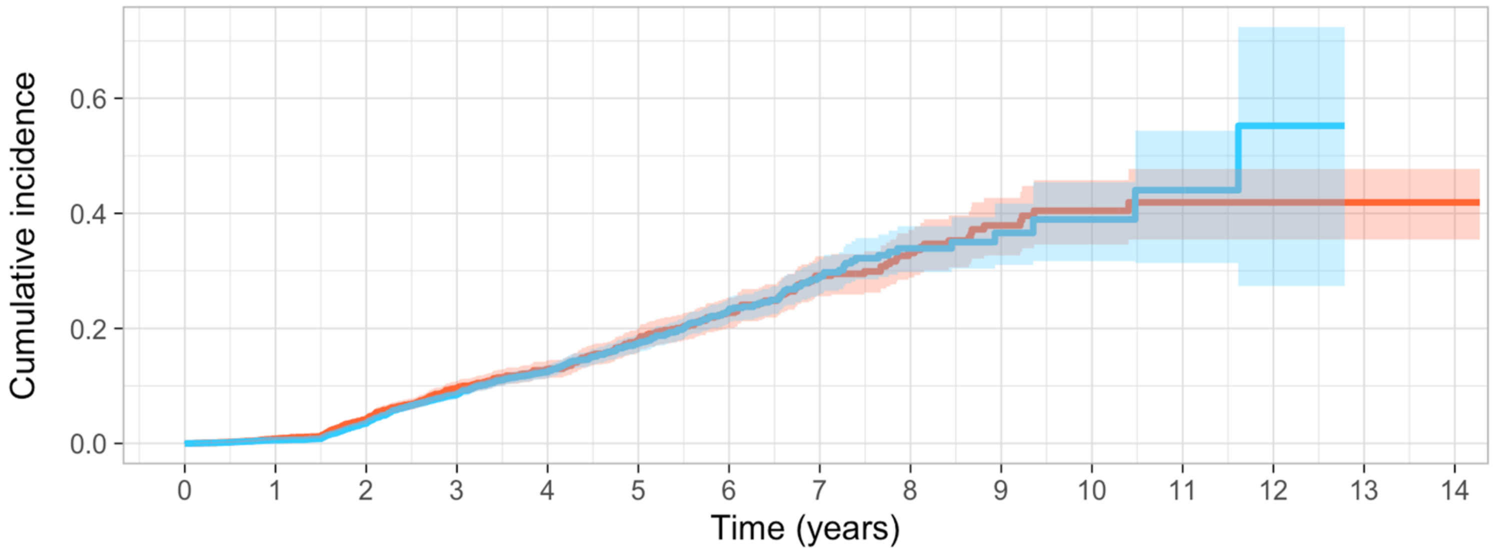

Number at risk

MET	5390	4059	2291	1351	872	556	361	220	133	84	53	24	<11	<11	<11
BUP	12635	9286	4950	2572	1543	885	467	220	90	36	16	<11	<11	<11	<11

Cumulative incidence

MET	0	0.01	0.04	0.1	0.13	0.18	0.23	0.29	0.33	0.38	0.4	0.42	0.42	0.42	0.42
BUP	0	0.01	0.03	0.09	0.12	0.17	0.23	0.29	0.34	0.37	0.39	0.44	0.55	0.55	0.55

Cumulative number of events

MET	0	39	134	243	283	325	352	376	385	393	396	397	397	397	397
BUP	0	61	242	449	542	613	660	685	696	698	699	700	701	701	701

Displayed are crude cumulative incidences of any neurodevelopmental disorders among children with prenatal exposure to methadone (MET, orange) or buprenorphine (BUP, blue) as estimated using the Kaplan-Meier estimator. Time of origin is delivery. Number at risk represent children under observation and event-free at the respective follow-up time.

Supplemental Figure S3. Cumulative incidence for individual neurodevelopmental disorders by exposure status

A) Attention Deficit Hyperactivity Disorder (ADHD)

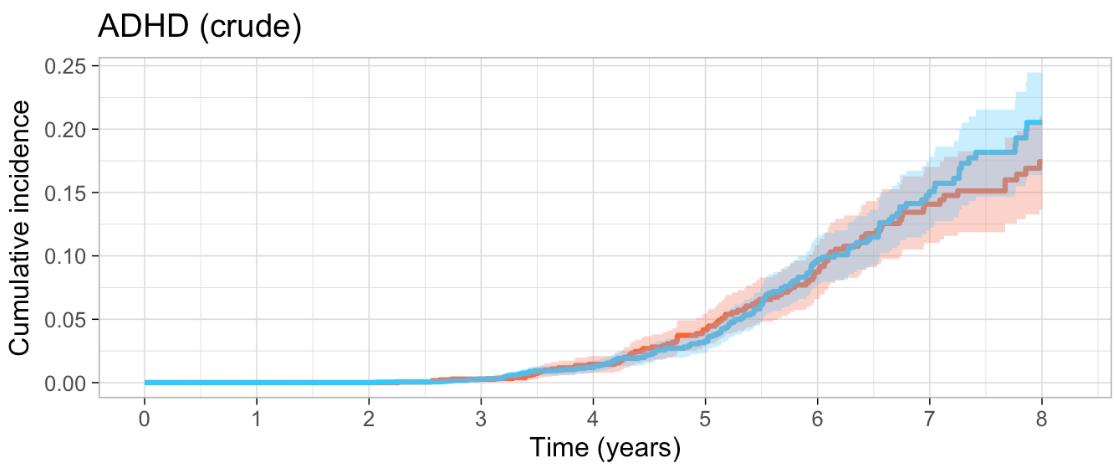

Number at risk

MET	5390	4090	2394	1500	980	648	426	264	160
BUP	12635	9341	5140	2811	1740	1021	549	264	115

Cumulative incidence

MET	0	0	0	0.003	0.014	0.042	0.087	0.141	0.174
BUP	0	0	0	0.003	0.012	0.033	0.096	0.151	0.205

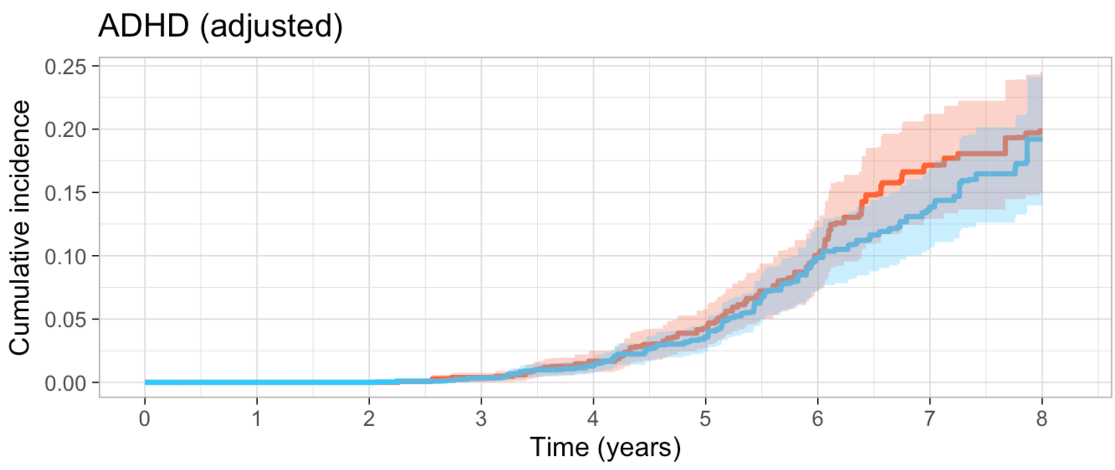

Number at risk

MET	2666	1987	1138	698	429	271	170	90	49
BUP	2666	1999	1121	644	404	248	140	77	40

Cumulative incidence

MET	0	0	0	0.004	0.017	0.043	0.1	0.172	0.199
BUP	0	0	0	0.003	0.013	0.036	0.098	0.138	0.192

Total cumulative number of Attention Deficit Hyperactivity Disorder events observed by age 8 years old was 147 in the buprenorphine and 96 in the methadone group.

B) Autism Spectrum Disorder (ASD)

ASD (crude)

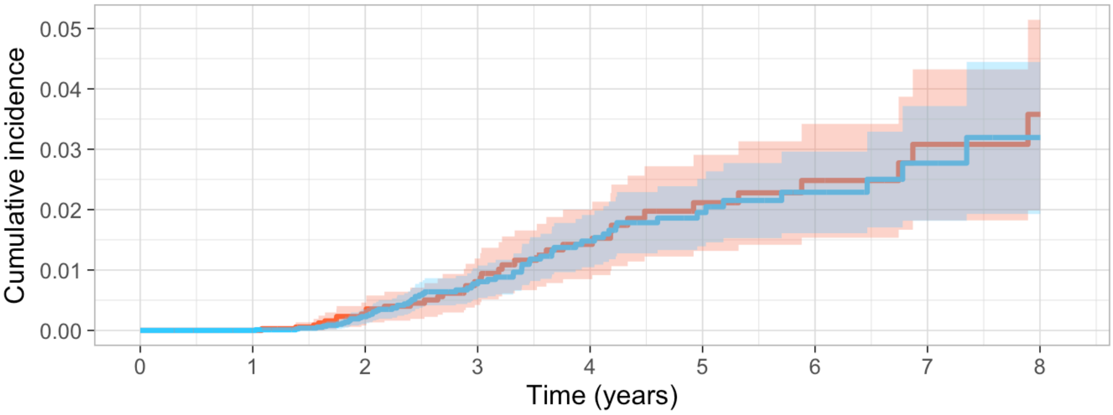

Number at risk

MET	5390	4090	2388	1492	981	669	459	301	188
BUP	12635	9341	5126	2799	1739	1044	600	302	141

Cumulative incidence

MET	0	0	0.003	0.008	0.014	0.021	0.025	0.031	0.036
BUP	0	0	0.002	0.008	0.015	0.02	0.023	0.028	0.032

ASD (adjusted)

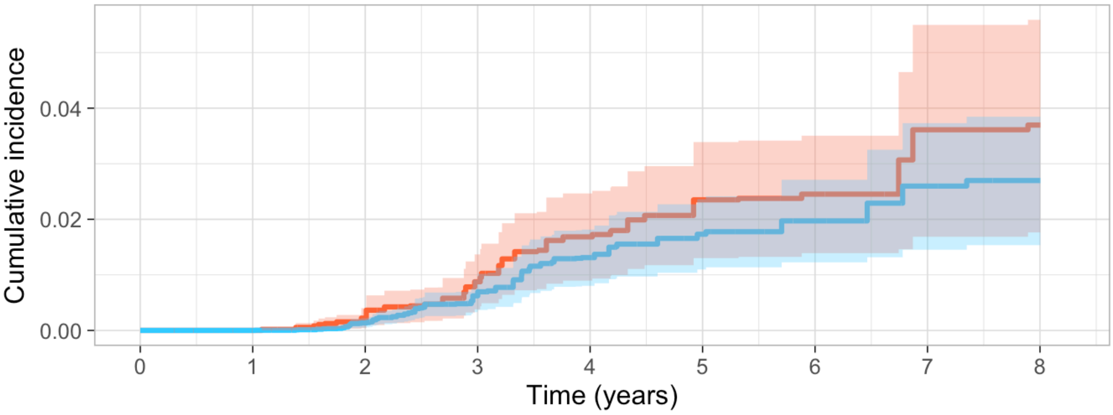

Number at risk

MET	2666	1987	1136	694	429	279	185	106	60
BUP	2666	1999	1119	643	405	255	153	86	47

Cumulative incidence

MET	0	0	0.002	0.009	0.017	0.024	0.025	0.036	0.037
BUP	0	0	0.001	0.007	0.013	0.017	0.02	0.026	0.027

Total cumulative number of autism spectrum disorder events observed by age 8 years old was 65 in the buprenorphine and 37 in the methadone group.

C) Behavioural Disorder

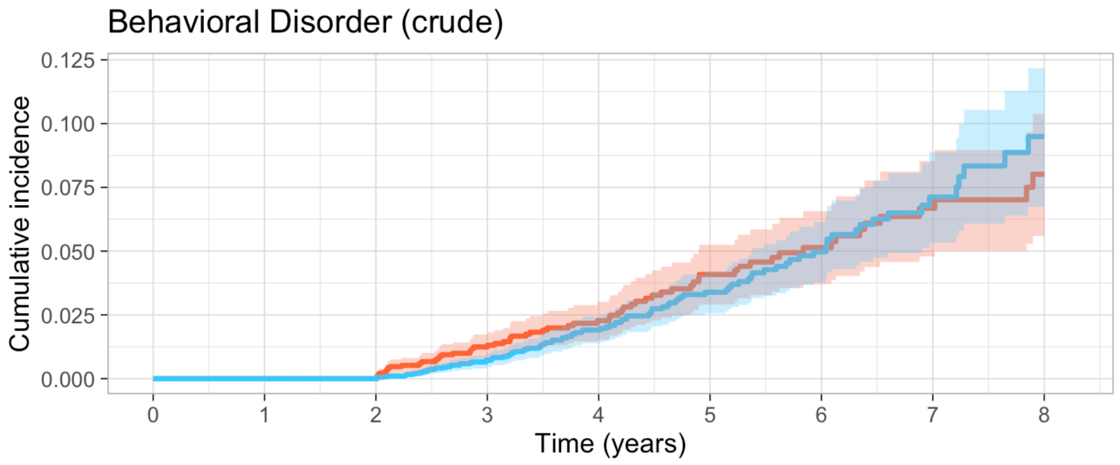

Number at risk

MET	5390	4090	2394	1482	970	646	437	282	174
BUP	12635	9341	5140	2800	1726	1026	580	282	129

Cumulative incidence

MET	0	0	0	0.013	0.023	0.041	0.051	0.067	0.08
BUP	0	0	0	0.007	0.019	0.034	0.05	0.071	0.095

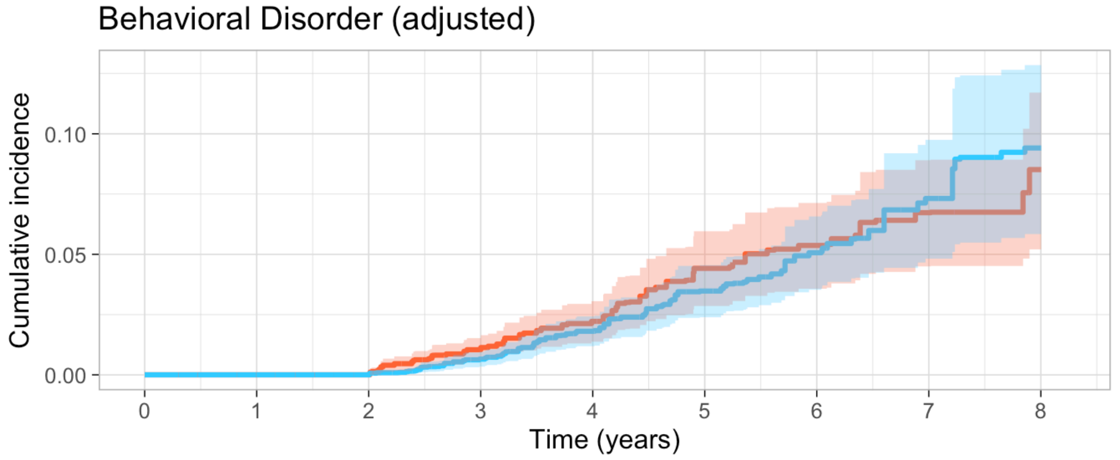

Number at risk

MET	2666	1987	1138	692	424	268	175	98	55
BUP	2666	1999	1121	642	402	249	148	81	43

Cumulative incidence

MET	0	0	0	0.011	0.022	0.044	0.054	0.067	0.085
BUP	0	0	0	0.007	0.018	0.035	0.051	0.073	0.094

Total cumulative number of behavioural disorder events observed by age 8 years old was 104 in the buprenorphine and 68 in the methadone group.

D) Developmental Coordination Disorder

Dev. Coordination Disorder (crude)

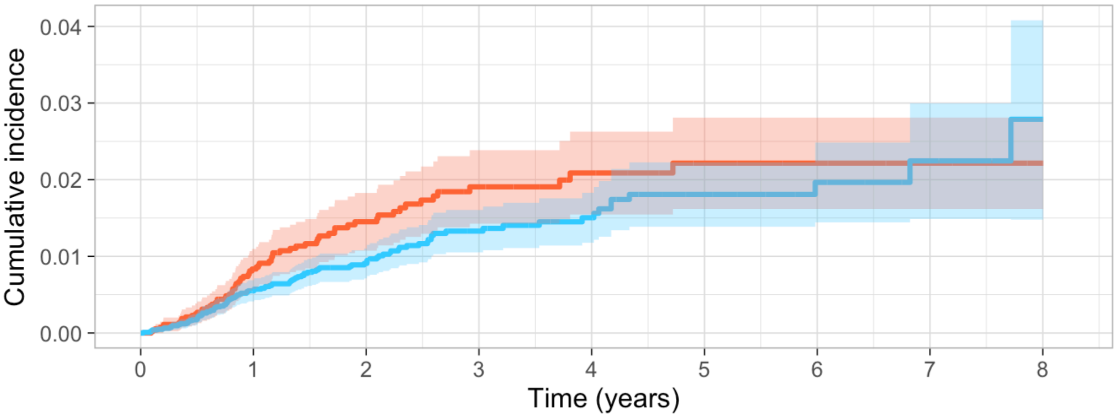

Number at risk

MET	5390	4059	2359	1484	986	675	466	305	193
BUP	12635	9286	5085	2788	1742	1055	610	304	142

Cumulative incidence

MET	0	0.008	0.015	0.019	0.021	0.022	0.022	0.022	0.022
BUP	0	0.006	0.009	0.013	0.015	0.018	0.02	0.022	0.028

Dev. Coordination Disorder (adjusted)

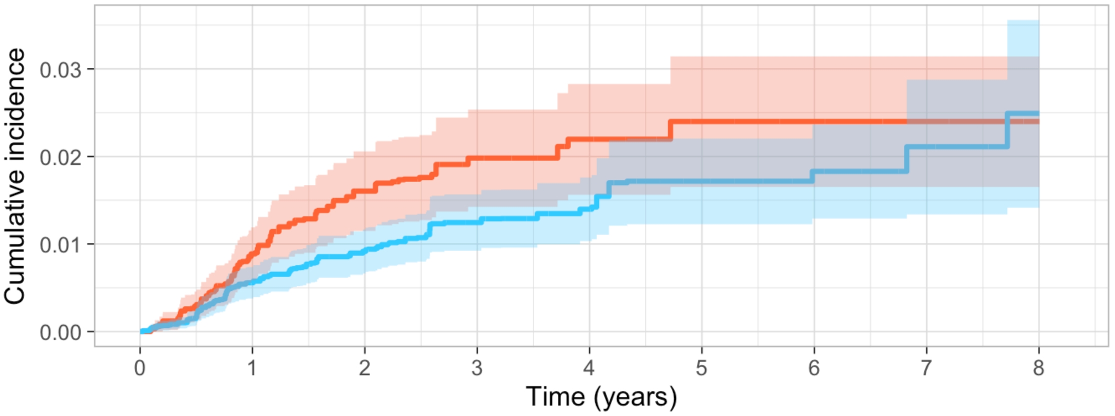

Number at risk

MET	2666	1972	1120	691	432	283	188	108	61
BUP	2666	1987	1109	640	406	258	156	87	48

Cumulative incidence

MET	0	0.009	0.016	0.02	0.022	0.024	0.024	0.024	0.024
BUP	0	0.006	0.009	0.012	0.014	0.017	0.018	0.021	0.025

Total cumulative number of developmental coordination disorder events observed by age 8 years old was 117 in the buprenorphine and 71 in the methadone group.

E) Developmental Speech/Language Disorder

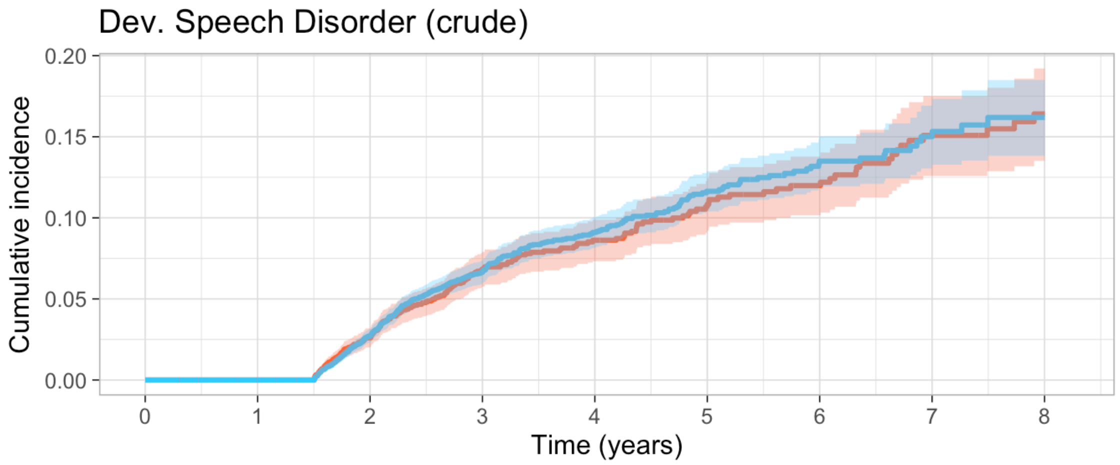

Number at risk

	0	1	2	3	4	5	6	7	8
MET	5390	4090	2326	1389	912	608	415	267	170
BUP	12635	9341	4997	2624	1603	954	537	265	120

Cumulative incidence

	0	1	2	3	4	5	6	7	8
MET	0	0	0.026	0.068	0.086	0.108	0.12	0.151	0.164
BUP	0	0	0.027	0.067	0.091	0.116	0.135	0.153	0.162

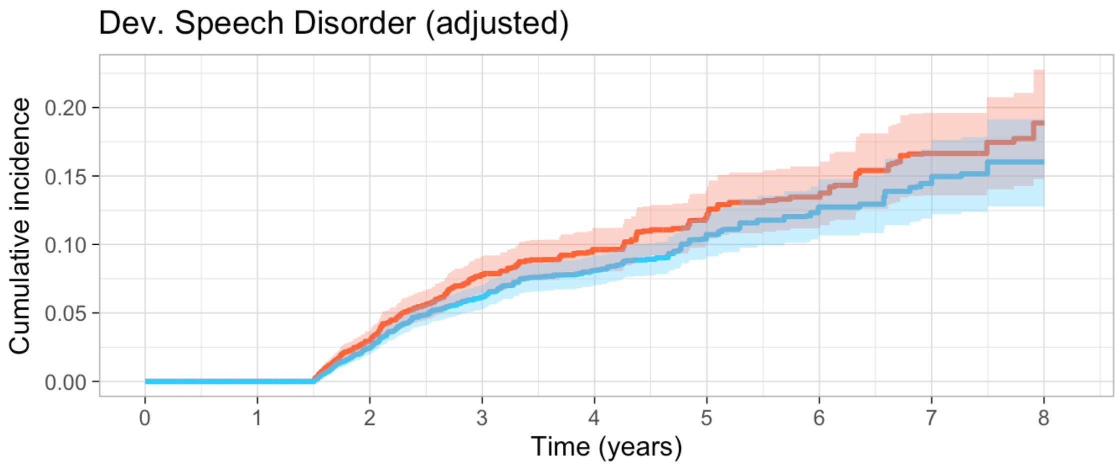

Number at risk

	0	1	2	3	4	5	6	7	8
MET	2666	1987	1101	640	395	249	164	91	53
BUP	2666	1999	1093	605	374	233	138	78	42

Cumulative incidence

	0	1	2	3	4	5	6	7	8
MET	0	0	0.029	0.078	0.096	0.122	0.135	0.167	0.189
BUP	0	0	0.025	0.062	0.081	0.107	0.127	0.15	0.16

Total cumulative number of developmental speech or language disorder events observed by age 8 years old was 446 in the buprenorphine and 218 in the methadone group.

F) Intellectual Disability

Intellectual Disability (crude)

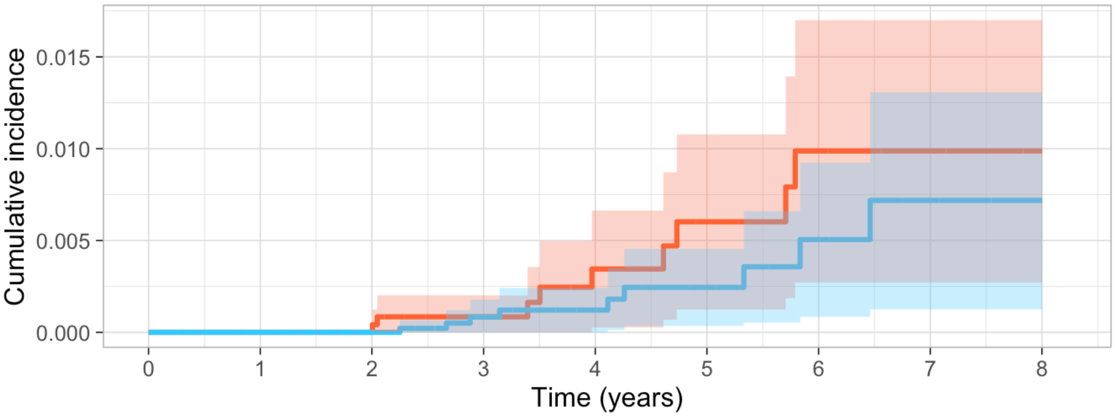

Number at risk

MET	5390	4090	2394	1503	992	676	465	302	190
BUP	12635	9341	5140	2819	1760	1062	611	306	143

Cumulative incidence

MET	0	0	0	0.001	0.003	0.006	0.01	0.01	0.01
BUP	0	0	0	0.001	0.001	0.002	0.005	0.007	0.007

Intellectual Disability (adjusted)

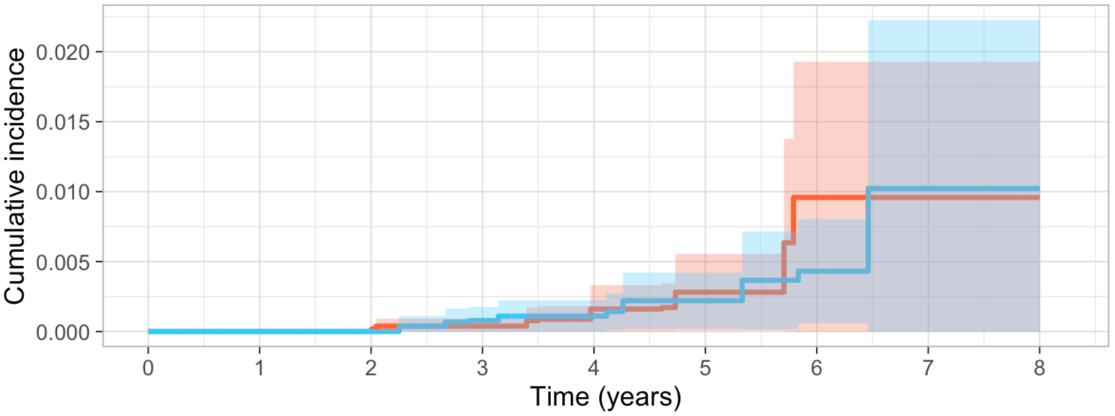

Number at risk

MET	2666	1987	1138	701	436	285	189	107	60
BUP	2666	1999	1121	647	410	260	156	87	48

Cumulative incidence

MET	0	0	0	0	0.002	0.003	0.01	0.01	0.01
BUP	0	0	0	0.001	0.001	0.002	0.004	0.01	0.01

Total cumulative number of intellectual disability events observed by age 8 years old was <11 in both the buprenorphine and the methadone group.

G) Learning Difficulty

Learning Difficulty (crude)

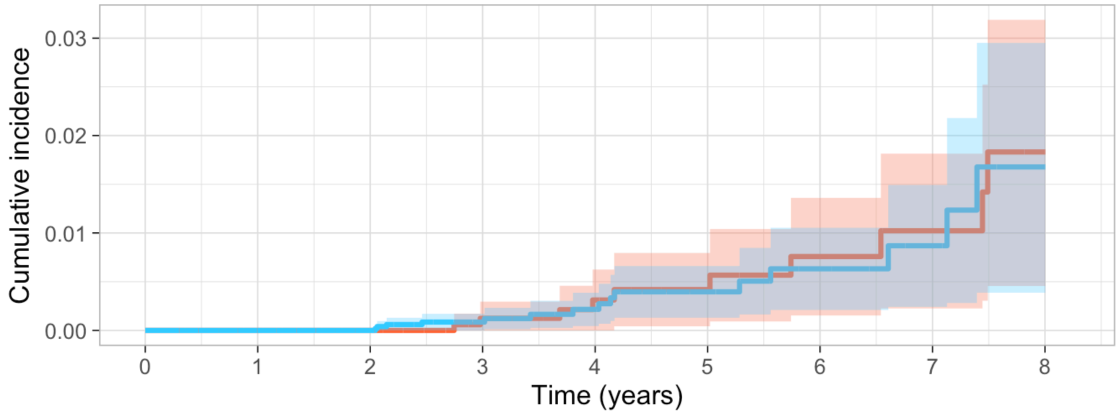

Number at risk

	0	1	2	3	4	5	6	7	8
MET	5390	4090	2394	1504	995	680	468	306	193
BUP	12635	9341	5140	2816	1758	1058	611	304	142

Cumulative incidence

	0	1	2	3	4	5	6	7	8
MET	0	0	0	0.001	0.003	0.004	0.008	0.01	0.018
BUP	0	0	0	0.001	0.002	0.004	0.006	0.009	0.017

Learning Difficulty (adjusted)

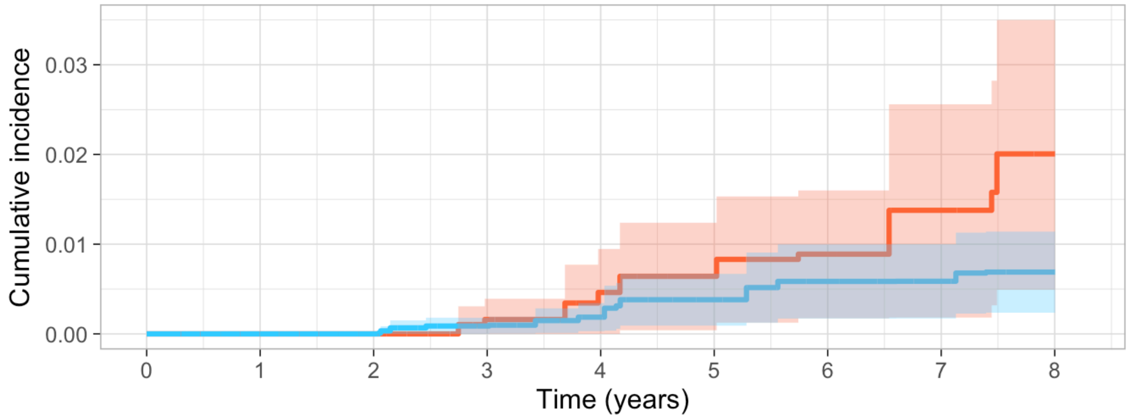

Number at risk

	0	1	2	3	4	5	6	7	8
MET	2666	1987	1138	701	437	285	190	109	62
BUP	2666	1999	1121	646	409	258	156	87	48

Cumulative incidence

	0	1	2	3	4	5	6	7	8
MET	0	0	0	0.002	0.005	0.006	0.009	0.014	0.02
BUP	0	0	0	0.001	0.002	0.004	0.006	0.006	0.007

Total cumulative number of learning difficulty events observed by age 8 years old was 15 in the buprenorphine and <11 in the methadone group.
